# Relation between Water Balance and Climatic Variables Associated with the Geographical Distribution of Anurans

**DOI:** 10.1371/journal.pone.0140761

**Published:** 2015-10-15

**Authors:** Braz Titon, Fernando Ribeiro Gomes

**Affiliations:** Departamento de Fisiologia, Instituto de Biociências, Universidade de São Paulo, São Paulo, São Paulo, Brazil; Trier University, GERMANY

## Abstract

Amphibian species richness increases toward the equator, particularly in humid tropical forests. This relation between amphibian species richness and environmental water availability has been proposed to be a consequence of their high rates of evaporative water loss. In this way, traits that estimate water balance are expected to covary with climate and constrain a species’ geographic distribution. Furthermore, we predicted that coexisting species of anurans would have traits that are adapted to local hydric conditions. We compared the traits that describe water balance in 17 species of anurans that occur in the mesic Atlantic Forest and xeric Cerrado (savannah) habitats of Brazil. We predicted that species found in the warmer and dryer areas would show a lower sensitivity of locomotor performance to dehydration (SLPD), increased resistance to evaporative water loss (REWL) and higher rates of water uptake (RWU) than species restricted to the more mesic areas. We estimated the allometric relations between the hydric traits and body mass using phylogenetic generalized least squares. These regressions showed that REWL scaled negatively with body mass, whereas RWU scaled positively with body mass. Additionally, species inhabiting areas characterized by higher and more seasonally uniform temperatures, and lower and more seasonally concentrated precipitation, such as the Cerrado, had higher RWU and SLPD than species with geographical distributions more restricted to mesic environments, such as the Atlantic Forest. These results support the hypothesis that the interspecific variation of physiological traits shows an adaptation pattern to abiotic environmental traits.

## Introduction

Amphibian species richness increases toward the equator, and higher diversity occurs in wet tropical forests, such as the Amazon Basin and the Atlantic Forest. This pattern in the distribution of species richness is attributed to two major abiotic factors, water availability and temperature [1]. In the literature, a commonly suggested functional cause for this association between patterns of amphibian species richness with water availability is high permeability of the skin, which is also an important organ of respiratory gas exchange [2,3].

In amphibians, dehydration affects both sprint and endurance performances, potentially reducing the ability to perform ecologically important behaviors, such as prey capture, escape from predators and search for mates [4]. Additionally, dehydration and body temperature have synergistic effects on locomotor performance [5]. Locomotor performance of both dehydrated and fully hydrated toads (*Anaxyrus americanus*) increases proportionally with the rise of body temperature, but maximum performance shifts toward lower temperatures in dehydrated individuals [5]. Additionally, some studies comparing a few species have pointed to a pattern of interspecific variation of this synergistic effect, with species found in open, hot and dry environments showing lower sensitivity of locomotor performance to dehydration at higher temperatures when compared with species from forested and wet environments [6–8].

Few comparative studies on amphibian water balance are based on a large number of species, and such studies have associated these physiological parameters with interspecific variation in habit or microhabitat use (arboreal, terrestrial, amphibious, and so forth) [9–12]. Regarding differences in microhabitat use, these studies have shown that arboreal frogs have high resistance to evaporative water loss and, consequently, lower rates of water loss than nonarboreal frogs [9,10]. Moreover, field studies have revealed that microhabitat use and season interact to determine interspecific variation in the hydric state of anurans. This pattern suggests that voluntary tolerance to dehydration varies with microhabitat use [11]. When active during the wet season, terrestrial species typically show a lower hydration state than arboreal and amphibious species, whereas amphibious species show a lower hydration state in natural refuges selected during the dry season when compared to terrestrial or arboreal species [11].

Additionally, dehydrated anurans uptake water through the pelvic patch, a specialized region of ventral skin characterized by high permeability and rich vascularization [13]. Rates of rehydration are reported to be higher for terrestrial species than aquatic ones and for species from arid environments when compared to species from mesic environments [14–17]. Other studies have also shown that, when compared to species from mesic environments, species that inhabit arid areas have higher levels of vascularization in the pelvic patch and a greater blood flow in this region when in contact with water after dehydration [18–22]. However, the limited number of species that have data on hydric balance precludes the ability to partition the roles of ecology versus phylogeny in shaping the evolution of rehydration rates.

The objective of this study was to investigate the evolution of water balance traits in anurans and the association of these traits with the climatic conditions that characterize the habitats found in their geographic distribution. We compared key traits associated with hydration state in a sample of anuran species that inhabit the Brazilian Atlantic Forest and the savannah area called the Cerrado. We predicted that species living in warmer and dryer areas would show a lower sensitivity of locomotor performance to dehydration, increased resistance to evaporative water loss and higher rates of water uptake than species from more mesic areas.

## Materials and Methods

### Collection Localities and Animal Maintenance

Males from 17 species of anurans were collected in several localities from Brazil (S1–S17 Figs). *Dendropsophus microps* (N = 7), *Scinax hayii* (N = 10), *S*. *crospedospillus* (N = 2), *Hypsiboas polytaenius* (N = 9), *H*. *faber* (N = 10), and *H*. *bischoffi* (N = 8) were collected at the Estação Biológica de Boracéia, Salesópolis, SP (23°39'13.99"S; 45°53'22.41"W) between 20 and 23 January 2009. *Leptodactylus notoaktites* (N = 3), *Physalaemus olfersii* (N = 7), *P*. *spiniger* (N = 8), *Proceratophrys boiei* (N = 10), and *S*. *rizibilis* (N = 10) were collected at the Parque Estadual Intervales, Ribeirão Grande, SP (24°16'24.55"S; 48°25'2.27"W) between 14 and 17 November 2009. *Rhinella ornata* (N = 9) were collected from an artificial pond at the University of São Paulo, São Paulo, SP (23°33'51.6"S; 46°43'48.1"W) between 23 and 24 October 2012. *Rhinella icterica* (N = 10) were collected at the countryside in São Luiz do Paraitinga, SP (23°10'06"S; 45°17'07"W) between 5 and 10 November 2013. Those collection sites belong to the Atlantic Forest Area [23]. *Hypsiboas albopunctatus* (N = 9), *D*. *minutus* (N = 10) and *L*. *podicipinus* (N = 7) were collected at the Estação Ecológica de Assis, Assis, SP (22°34'19.88"S; 50°24'32.82"W) between 5 and 8 March 2009. *Rhinella schneideri* (N = 9) were collected at the countryside in Luiz Antônio, SP (21°33'05"S; 47°39'16"W) between 25 and 27 February 2013. Those last two sites belong to the Cerrado area [23]. After collection, the animals were brought to the laboratory in the University of São Paulo and kept in individual plastic containers, where they were exposed to the natural light/dark cycles and temperature and provided with freely available water and some type of shelter. They were fed cockroaches once per week. The measurements were performed in sequence (resistance to evaporative water loss, sensitivity of locomotor performance to dehydration and rates of water uptake) for up to 21 days after the animals arrived at the laboratory. When individuals did not appear visually healthy, the next measurements were cancelled. Consequently, the number of species for which we have data differ for the physiological variables. The animals were collected under license for capture and transport from the “Instituto Brasileiro do Meio Ambiente e dos Recursos Naturais Renováveis” (IBAMA, process numbers 17377–1 and 29896–1), and procedures for the collection and use of biological material were performed with the approval of the “Comissão de Ética na Experimentação Animal”, process number 62/08-CEEA, UNESP—Univ Estadual Paulista, Botucatu, Biosciences Institute and “Comissão de Ética no Uso de Animais”, process number 120/2010-CEUA, Biosciences Institute, University of São Paulo. Field work at Estação Ecológica de Assis and Parque Estadual Intervales were conducted under authorization of the “Coordenadoria de informações técnicas, documentação e pesquisa ambiental” (COTEC, process number 260108–000.000.002.011/0 2008), Instituto Florestal, Secretaria do Meio Ambiente. For the other localities, no specific authorization was required.

### Sensitivity of locomotor performance to dehydration

Locomotor tests were performed at 40% RH, 25°C and five consecutive levels of hydration (100, 90, 80, 75 and 70% of the standard body mass), and the animals were maintained inside an environmental chamber with temperature and humidity controls (FITOTRON 011 –Eletrolab, São Paulo, São Paulo, Brazil). Prior to testing, animals were maintained in plastic containers (0.13 m×0.13 m×0.11 m) filled with tap water for 1 h at the test temperature. They were then carefully blotted with paper tissue, their bladders were emptied by gently pressing their abdomens, and their body masses were recorded (±0.0001 g). This body mass was considered as the standard mass (hydration level of 100%). The animals were stimulated to jump on the floor of the Fitotron by tapping it gently for six consecutive times. Starting and landing points were marked on the floor, and later, the distance between marks was measured to the nearest 1 cm. The longest jump of the series was used as the best estimate of maximum jumping performance. Anurans were subsequently dehydrated and locomotor performance tests were performed each time the individuals reached one of the intended hydration levels. The evaporative water loss was controlled in order to maintain intervals of 60 min between the locomotor tests. Electric fans were used to accelerate the rates of evaporative water loss when necessary. Consecutive tests were performed until anurans reached 75% or 70% of the standard body mass, depending on their general condition and responsiveness to stimuli. Given that toads are characterized by high aerobic locomotor capacity [8,24,25], locomotor performance for *Rhinella* was measured as the distance moved in a circular track (1.5 m diameter) during 10 minutes at each hydration condition. For these animals, consecutive tests of performance at different hydration levels were performed on different days, resulting in a dehydration rate of 10% a day until 80%, and then 5% a day until individuals reached 75% or 70% of the standard body mass. For each individual, graphs were built with the hydration level on the x-axis and the locomotor performance (corrected by the snout-vent length) on the y-axis (S18–S30 Figs). Sensitivity of locomotor performance to dehydration (SLPD) was visually interpolated from these graphs and considered as the hydration state that results in 70% of maximum performance [6,8].

### Resistance to evaporative water loss

Prior to measuring evaporative water loss, we maintained the anurans in plastic containers (0.13 m×0.13 m×0.11 m) filled with tap water during 1 h at the test temperature. They were then carefully blotted in paper tissue, their bladders were emptied by gently pressing their abdomen and their body masses were recorded (±0.01 g). Animals were then individually put into clear acrylic chambers based on the size of the species (140 mm diameter × 110 mm high for larger animals and 60 mm diameter X 55 mm high for smaller ones) at 25°C. An open flow system was used to measure the rates of evaporative water loss and the resistance to evaporative water loss. Flow rates of 23 cm^3^ s^-1^ for the larger chambers and 5 cm^3^ s^-1^ for the smaller ones were generated by a set of air pumps connected to a mass flowmeter (SS-3 Subsampler—Sable Systems, Las Vegas, Nevada, USA) that allowed the same flux for each chamber to be sent individually. Air pumped to the chambers was maintained at a relative humidity of 20% by using a humidity controller (RH/Dewpoint Controller—Sable Systems, Las Vegas, Nevada, USA). At each measurement, airflow passed through three chambers: one empty chamber, one chamber containing a 3% agar model with size and shape approximated to the size and shape of each species, and one chamber containing the animal. The air leaving each chamber was sent to an 8-channel multiplexer (RM8-Intelligent Multiplexer—Sable Systems, Las Vegas, Nevada, USA) and then to a vapor density analyzer (RH-300 RH/Dewpoint Analyzer—Sable Systems, Las Vegas, Nevada, USA). An interface (UI-2 Data Acquisition Interface—Sable Systems, Las Vegas, Nevada, USA) connected to a computer allowed continuous data recording. Only records corresponding to periods when the animals stayed in a water conservation posture, identified by visual monitoring and constancy of the water vapor density values, were considered for calculations. After maintaining a stable vapor density value for at least 20 minutes, the chambers were opened and the surface temperature of both the animal and the agar model was measured by an infrared thermometer (TR-300 –Equitherm, São Paulo, São Paulo, Brazil).

The rates of evaporative water loss were calculated from the formula:

EWL=FaVDa−FeVDe

where *EWL* means absolute rates of evaporative water loss (μg s^-1^), *Fa* means airflow (cm^3^ s^-1^) through the chamber with the animal or the agar model, *Fe* means air flow (cm^3^ s^-1^) through the empty chamber, *VDa* means water vapor density (μg cm^-3^) from the chamber with the animal or the agar model, and *VDe* means water vapor density (μg cm^-3^) from the empty chamber. Cutaneous rates of water loss by area (*CWL*) were calculated by dividing the absolute rates by 2/3 of the total surface area, which correspond to the area exposed to air when anurans keep the water conservation posture [26]. Surface area was estimated using the following formula [27]:

$$SA=9.9M^{0.56}$$

where *SA* means surface area (cm^2^) and *M* means body mass (g). Finally, total resistance to evaporative water loss was calculated from the following formula:

r=VDD/CWL

where *r* means the resistance to evaporative water loss (s cm^-1^), *VDD* means the difference between the saturated water vapor density (μg cm^-3^) at the surface of the animal or the agar model, and the air leaving the chamber containing the animal or the agar model and *CWL* means cutaneous rates of evaporative water loss by area (μg s^-1^ cm^-2^) for the animal or the agar model. Saturated water vapor density at the surface of the animal or the agar model was calculated from the ideal gas law using the surface temperature. The resistance to evaporative water loss calculated for the animal and the agar model correspond, respectively, to the total resistance to evaporative water loss and the resistance to evaporative water loss of the boundary layer. By subtracting the boundary layer resistance from the total resistance to evaporative water loss, we obtained the skin resistance to evaporative water loss (REWL) [28].

### Rates of water uptake

Dehydrated anurans (approximately 70% of the standard body mass) were maintained in individual containers filled with water at a depth sufficient to cover only the ventral region of the animal. The animals were taken, carefully blotted with paper tissue and weighed (± 0.0001 g) every 2 minutes for 6 consecutive times. The rates of water uptake were calculated from the regression of the body mass gain against time and expressed as (μg s^-1^). Rates of water uptake by area (RWU) were calculated by dividing the absolute rates by 1/3 of the total surface area, corresponding to the ventral surface area in contact with water during rehydration [26]. Although the animals were weighed every 2 minutes to obtain the estimates of RWU, they remained motionless during the tests, and the curves of body mass gain by time presented R^2^ values higher than 0.9.

### Species occurrence and climatic data

Geographical coordinates of occurrence for each species were compiled from the speciesLink Project (S1–S17 Figs) [29]. For each coordinate, mean data from 1950 to 2000 on eight climatic variables (annual mean temperature, maximum temperature of the warmest month, minimum temperature of the coldest month, temperature seasonality, annual precipitation, precipitation of the wettest month, precipitation of the driest month and precipitation seasonality) were extracted from Worldclim with 30 arc-seconds resolution [30,31] using DIVA-GIS [32] version 7.5.0.0.

### Phylogenetic relationships

**Fig 1:**
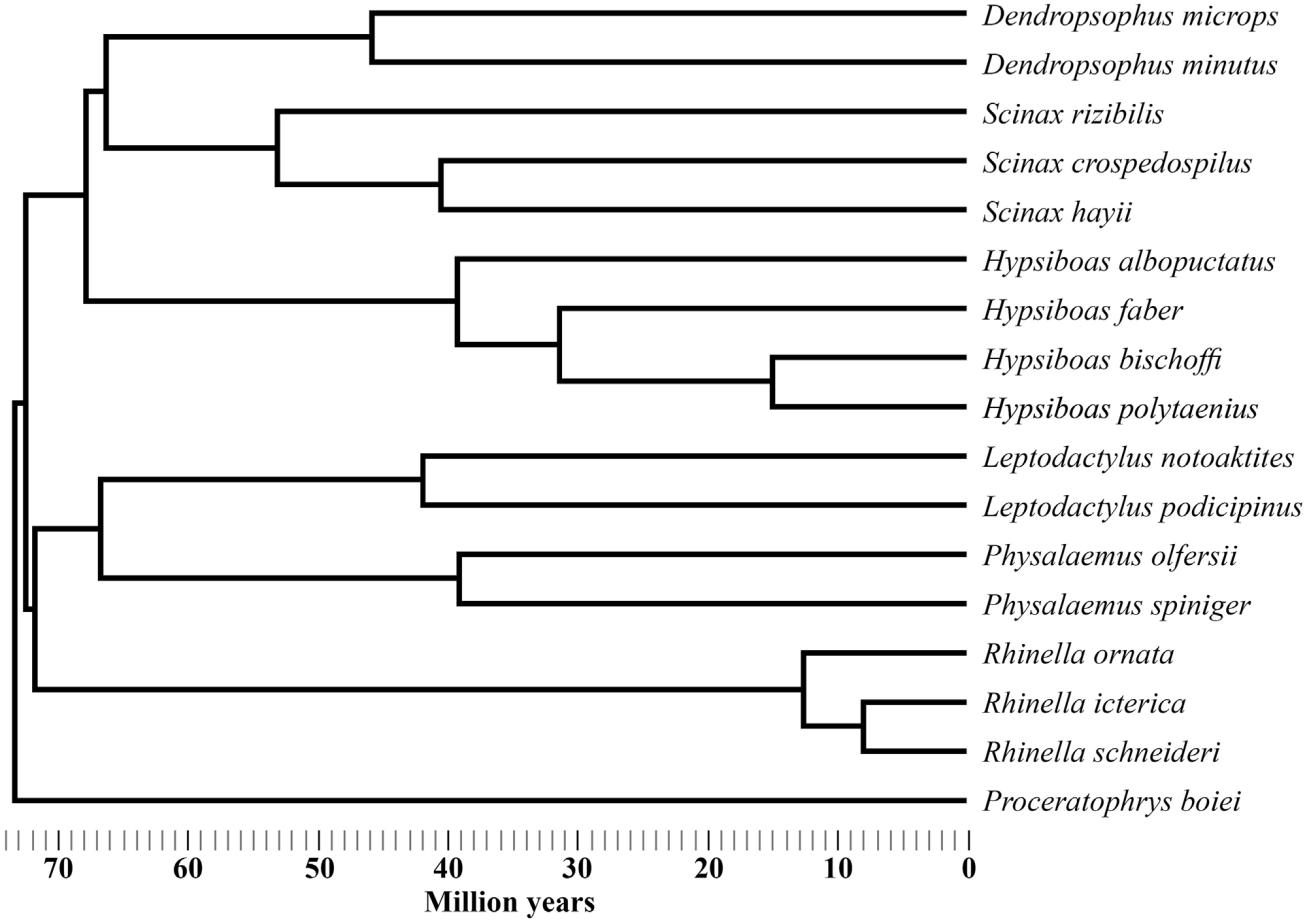
Phylogenetic tree. Composite phylogenetic tree for the 17 anuran species included in the present study, with topology and divergence times based on the literature [33–35].

A composite tree with divergence time estimates for the 17 species of anurans was compiled (Fig 1), mainly based on [33], which is the most comprehensible current phylogenetic hypothesis for the anurans. Phylogenetic information that was not available in [33] was gathered from [34,35], as follows: to include *D*. *microps*, a species from the *D*. *parveceps* group of species [35], the topological position and divergence time for *D*. *parveceps* in [33] was assumed; to include *S*. *hayii*, a species that is within a polytomy that includes *S*. *fuscovarius* in [34], the topological position and divergence time for *S*. *fuscovarius* in [33] was assumed; and to include *S*. *rizibilis*, a species from the clade that includes *S*. *berthae* in [34], the topological position and divergence time of *S*. *berthae* in [33] was assumed. Finally, *P*. *olfersii* and *P*. *spiniger* were inserted in the phylogeny considering the maximum time of divergence from this genus in [33]. The phylogenetic information used to build this composite tree was primarily gathered from molecular studies. For the few instances in which morphological data were employed [34], these were probably not related to the physiological variables investigated in the present study. Consequently, the information used to build this composite tree and the data analyzed in the present study were confidently independent. There were no topological divergences between the phylogenetic proposals used to build our composite tree. The number of species included in the study differed for some physiological measurements. Consequently, the trees used to analyze the relations between climatic variables and physiological variables had 16 species for REWL and 13 species for RWU and SLPD.

### Statistical analyses

Descriptive statistics were performed for all physiological and climatic data per species, and data were posteriorly transformed to Log_10_ for subsequent analyses. For each species, means of the eight climatic variables extracted from each locality were implemented in principal component analyses (PCA), and the scores from the components with eigenvalues greater than 1.0 were saved for *a posteriori* analyses. We considered any absolute values higher than 0.65 as a high load. Again, given that the number of species included in the study differed for some physiological measurements, two PCAs were conducted: one containing climatic data for the 16 species from which there were data on REWL, and another containing climatic data for the 13 species from which data on RWU and SLPD were available.

Phylogenetic regressions were used to investigate the relationship between the physiological variables and body mass [36], and the residuals of data phylogenetic corrected by size were saved to be implemented in *a posteriori* analyses. Phylogenetic regressions [37] were employed to investigate the relationships between physiological variables and climatic data. Physiological variables corrected by size (SLPD, REWL and RWU) were entered into the regression models as dependent variables, and two components from the PCA of climatic variables with eigenvalues higher than 1.0 were entered as predictors. Additionally, a phylogenetic ANOVA was implemented using the biome where individuals from the different species were collected for physiological measurements (Atlantic Forest and the Cerrado) as a categorical factor.

Descriptive statistics and principal component analyses of the climatic variables were performed using the software SPSS for Windows version 13.0. Phylogenetic trees were built using Mesquite version 2.75 (build 564). Procedures for phylogenetic size-correction, phylogenetic regressions and phylogenetic ANOVA were conducted with the software R version 3.0.2 (2013-09-25). The phylogenetic regressions were performed using the function gls, from the package nlme to fit a linear model using generalized least squares. The function corPagel from the package ape, was used to determine the structure of Pagel's “lambda” correlation. The comparison between sites of collection in the Atlantic Forest and the Cerrado was performed using the function phylANOVA, from the package phytools, with 1000 simulations and Bonferroni correction.

## Results

Descriptive statistics for phenotypic variables (body mass, REWL, RWU and SLPD) from each species are presented in Table 1 as the mean ± standard deviation. Descriptive statistics for climatic variables from the localities of species occurrence related to temperature (annual mean temperature, maximum temperature of the warmest month, minimum temperature of the coldest month and temperature seasonality) and precipitation (annual precipitation, precipitation of the wettest month, precipitation of the driest month and precipitation seasonality) are presented, respectively, in Tables 2 and 3 as the mean ± standard deviation.

**Table 1:** Mean ± standard deviation of the phenotypic variables collected for 17 species of anurans.

Species	N	Body Mass	REWL	RWU	SLPD
		(g)	(s cm^-1^)	(μg cm^-2^ s^-1^)	(%)
*Dendropsophus microps*	7	0.55 ± 0.03	9.23 ± 0.93	27.80 ± 4.38	76.5 ± 4.3
*Dendropsophus minutus*	10	0.50 ± 0.03	-	56.59 ± 19.82	83.5 ± 4.0
*Scinax rizibilis*	10	0.72 ± 0.07	3.76 ± 1.44	-	-
*Scinax crospedospilus*	2	1.25 ± 0.01	3.13 ± 0.01	-	-
*Scinax hayii*	10	3.51 ± 0.41	4.42 ± 0.74	57.45 ± 12.84	83.8 ± 3.6
*Hypsiboas albopuctatus*	9	5.86 ± 0.91	2.54 ± 0.72	65.62 ± 20.04	83.7 ± 5.8
*Hypsiboas faber*	10	38.25 ± 5.09	3.34 ± 0.63	95.42 ± 22.10	76.9 ± 2.1
*Hypsiboas bischoffi*	8	3.50 ± 0.49	5.60 ± 1.57	91.61 ± 22.10	74.0 ± 1.6
*Hypsiboas polytaenius*	10	1.12 ± 0.11	5.46 ± 1.86	55.23 ± 20.50	78.5 ± 2.1
*Proceratophrys boiei*	10	11.96 ± 1.87	2.35 ± 0.39	128.36 ± 25.17	77.1 ± 1.6
*Leptodactylus notoaktites*	3	12.09 ± 1.13	2.53 ± 0.60	-	-
*Leptodactylus podicipinus*	7	4.46 ± 1.57	1.70 ± 0.35	33.26 ± 6.74	84.1 ± 3.5
*Physalaemus olfersii*	7	2.96 ± 0.65	2.85 ± 0.46	20.41 ± 4.70	81.2 ± 4.6
*Physalaemus spiniger*	8	0.50 ± 0.08	2.63 ± 0.62	-	-
*Rhinella ornata*	9	15.20 ± 3.00	4.43 ± 0.51	76.18 ± 23.80	81.7 ± 2.1
*Rhinella icterica*	10	94.85 ± 21.20	1.16 ± 0.28	182.80 ± 56.09	81.3 ± 4.0
*Rhinella schneideri*	9	78.48 ± 21.35	1.96 ± 0.71	223.55 ± 56.77	79.2 ± 2.4

N: number of individuals used for data collection; REWL: resistance to evaporative water loss; RWU: rates of water uptake; SLPD: sensitivity of locomotor performance to dehydration, considered as the hydration state that results in 70% of maximum performance.

**Table 2:** Mean ± standard deviation of the climatic variables related to temperature extracted from occurrence data for 17 species of anurans.

Species	N	AMT	MTWM	MTCM	TS
		(°C)	(°C)	(°C)	(°C)
*Dendropsophus microps*	55	18.1 ± 2.2	26.5 ± 2.2	8.6 ± 2.7	2.7 ± 0.4
*Dendropsophus minutus*	579	21.9 ± 2.9	30.2 ± 2.7	12.2 ± 3.4	1.9 ± 0.9
*Scinax rizibilis*	39	18.8 ± 2.0	27.6 ± 2.0	9.1 ± 2.1	2.9 ± 0.3
*Scinax crospedospilus*	31	18.8 ± 2.1	26.8 ± 2.2	9.2 ± 2.4	2.4 ± 0.3
*Scinax hayii*	76	19.1 ± 2.3	26.9 ± 2.4	9.7 ± 2.9	2.4 ± 0.3
*Hypsiboas albopuctatus*	540	21.7 ± 2.3	29.8 ± 2.3	11.5 ± 2.5	2.0 ± 0.6
*Hypsiboas faber*	169	20.1 ± 2.3	28.5 ± 2.0	10.8 ± 3.2	2.5 ± 0.5
*Hypsiboas bischoffi*	59	17.9 ± 1.9	26.6 ± 1.8	8.3 ± 2.1	2.9 ± 0.3
*Hypsiboas polytaenius*	33	19.0 ± 2.2	26.9 ± 2.2	9.2 ± 2.9	2.2 ± 0.2
*Proceratophrys boiei*	121	18.6 ± 2.0	26.7 ± 2.1	9.2 ± 2.6	2.5 ± 0.4
*Leptodactylus notoaktites*	29	19.2 ± 2.1	28.0 ± 2.1	9.4 ± 2.2	3.0 ± 0.3
*Leptodactylus podicipinus*	283	23.4 ± 1.6	31.3 ± 1.6	13.0 ± 2.6	1.9 ± 0.6
*Physalaemus olfersii*	77	18.1 ± 1.9	26.3 ± 2.2	8.6 ± 2.0	2.6 ± 0.3
*Physalaemus spiniger*	16	21.8 ± 0.9	30.3 ± 0.8	12.6 ± 1.4	3.0 ± 0.2
*Rhinella ornata*	113	20.2 ± 1.9	28.1 ± 2.0	10.7 ± 2.4	2.5 ± 0.3
*Rhinella icterica*	163	18.6 ± 2.2	27.0 ± 2.3	9.1 ± 2.4	2.7 ± 0.4
*Rhinella schneideri*	143	22.8 ± 1.9	30.7 ± 1.9	12.4 ± 2.3	2.0 ± 0.5

N: number of occurrence points obtained for each species; AMT: annual mean temperature; MTWM: maximum temperature of warmest month; MTCM: minimum temperature of coldest month; TS: temperature seasonality (considered as the standard-deviation of monthly mean temperature).

**Table 3:** Mean ± standard deviation of the climatic variables related to precipitation extracted from occurrence data for 17 species of anurans.

Species	N	AP	PWM	PDM	PS
		(mm)	(mm)	(mm)	(mm)
*Dendropsophus microps*	55	1586 ± 372	231 ± 52	58 ± 25	47 ± 15
*Dendropsophus minutus*	579	1489 ± 295	245 ± 54	32 ± 35	64 ± 24
*Scinax rizibilis*	39	1514 ± 330	213 ± 43	61 ± 19	42 ± 12
*Scinax crospedospilus*	31	1624 ± 420	255 ± 46	44 ± 25	59 ± 12
*Scinax hayii*	76	1765 ± 413	271 ± 44	51 ± 26	57 ± 13
*Hypsiboas albopuctatus*	540	1411 ± 243	250 ± 47	22 ± 22	72 ± 17
*Hypsiboas faber*	169	1516 ± 353	222 ± 49	55 ± 33	49 ± 21
*Hypsiboas bischoffi*	59	1612 ± 358	217 ± 42	72 ± 32	39 ± 17
*Hypsiboas polytaenius*	33	1593 ± 362	273 ± 47	34 ± 27	68 ± 17
*Proceratophrys boiei*	121	1547 ± 272	245 ± 47	47 ± 20	56 ± 15
*Leptodactylus notoaktites*	29	1462 ± 202	212 ± 34	56 ± 17	45 ± 13
*Leptodactylus podicipinus*	283	1345 ± 216	236 ± 35	18 ± 10	72 ± 11
*Physalaemus olfersii*	77	1614 ± 308	246 ± 47	53 ± 18	52 ± 11
*Physalaemus spiniger*	16	1856 ± 391	269 ± 66	69 ± 15	45 ± 5
*Rhinella ornata*	113	1640 ± 430	249 ± 45	50 ± 24	56 ± 11
*Rhinella icterica*	163	1653 ± 326	234 ± 50	65 ± 32	45 ± 19
*Rhinella schneideri*	143	1368 ± 225	237 ± 40	21 ± 14	69 ± 12

N: number of occurrence points obtained for each species; AP: annual precipitation; PWM: precipitation of wettest month; PDM: precipitation of driest month; PS: precipitation seasonality (considered as the coefficient of variation of monthly mean precipitation).

### Relations between climatic variables

The principal component analyses performed on climatic variables retained two components, which were the same when using a data set of 16 or 13 species (Table 4). Component 1 explained 66.36% and 75.68% of the total variance observed for the sets of 16 and 13 species, respectively, and represents a direct association between the annual mean temperature, maximum temperature of the warmest month, minimum temperature of the coldest month and precipitation seasonality, which are inversely associated with the temperature seasonality, annual precipitation and precipitation of driest month. Component 2 explained 19.87% and 18.99% of the total variance observed for the sets of 16 and 13 species, respectively, and is mainly associated with precipitation of the wettest month.

**Table 4:** Results from two principal component analyses performed on climatic variables extracted from points of occurrence of Brazilian anuran species.

Climatic variables	16 species*		13 species*	
	C1	C2	C1	C2
Annual Mean Temperature	**0.883**	-0.062	**0.976**	-0.126
Max Temperature of Warmest Month	**0.812**	-0.212	**0.954**	-0.256
Min Temperature of Coldest Month	**0.938**	-0.192	**0.948**	-0.145
Temperature Seasonality	**-0.921**	-0.135	**-0.961**	-0.112
Annual Precipitation	**-0.677**	0.593	**-0.856**	0.383
Precipitation of Wettest Month	0.239	**0.966**	0.169	**0.982**
Precipitation of Driest Month	**-0.96**	-0.089	**-0.969**	-0.132
Precipitation Seasonality	**0.839**	0.44	**0.818**	0.525
Eigenvalues	5.309	1.59	6.054	1.519
% of Variance Explained	66.36	19.87	75.68	18.99

C1: component 1; C2: component 2;

*: two principal component analyses performed for sets of 16 species and 13 species, according to physiological data available for them. Values with higher loadings (≥0.65) in each component are highlighted in boldface.

### Allometry and relations between climate and physiological variables

**Fig 2:**
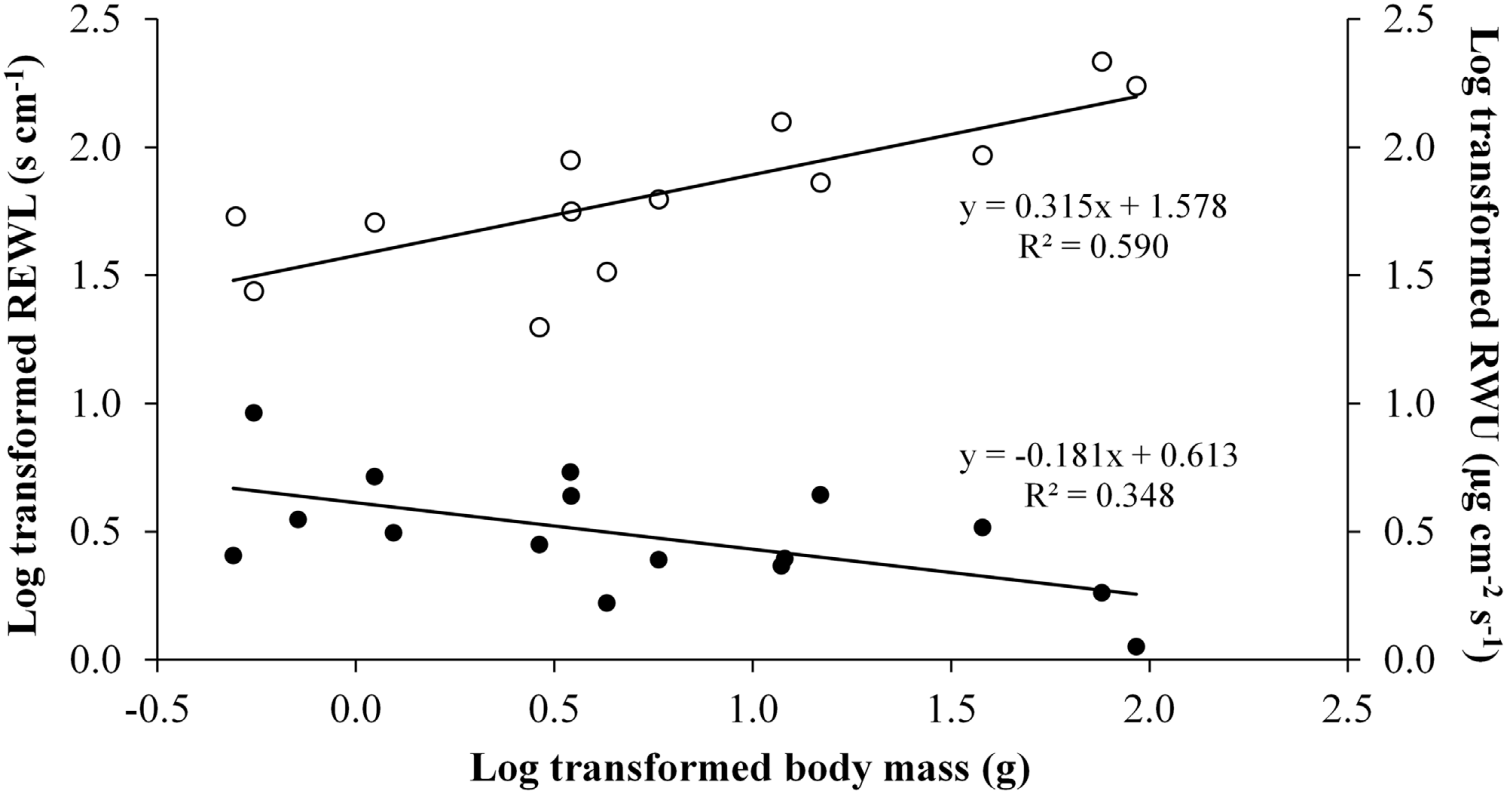
Allometric relations between physiological variables and body mass. Regressions of resistance to evaporative water loss (REWL) and rates of water uptake (RWU) as functions of body mass (BM). The regression line equations stated in the figure represent the conventional linear regression. The equations from phylogenetic regressions are REWL = -0.185BM + 0.608 and RWU = 0.327BM + 1.538. Fulfilled circles represent the mean resistance to evaporative water loss for each species and unfilled circles represent the mean rate of water uptake for each species.

The REWL declined as the body mass increased (REWL = 0.608BM^-0.185^, *p* = 0.021). In contrast, RWU increased with body mass (RWU = 1.538BM^0.327^, *p* = 0.004). Thus, large species showed a lower REWL but had a higher RWU (Fig 2). A relation between body mass and SLPD was not observed (SLPD = 1.909BM^-0.005^, *p* = 0.584).

**Fig 3:**
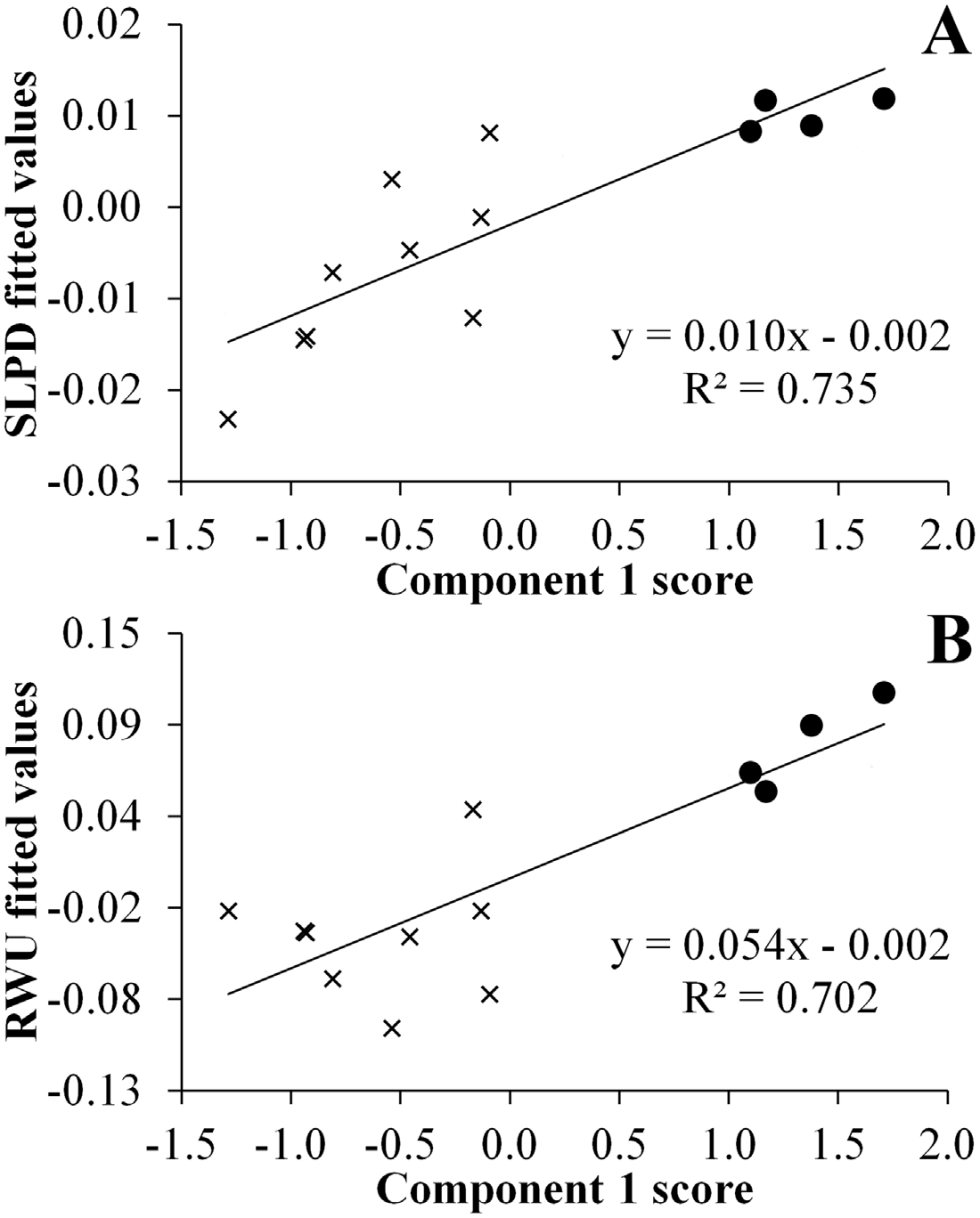
Phylogenetic relations between physiological and climatic variables. Relations between size adjusted sensitivity of locomotor performance to dehydration (A) and rates of water uptake (B) with the PC scores from axis 1. The phylogenetically corrected values for the physiological traits show a positive correlation with the PCA component 1 scores that correspond to a direct association between annual mean temperature, maximum temperature of warmest month, minimum temperature of coldest month and precipitation seasonality, which are inversely associated with temperature seasonality, annual precipitation and precipitation of driest month. Fulfill circles represent species collected at the Cerrado sites and “X” represent species collected at Atlantic Forest sites.

SLPD and RWU were directly affected by component 1 of the climatic PCA (Table 5). Interspecific variance in anuran REWL showed no association with the climatic components (Table 5). RWU showed a higher phylogenetic signal when compared to REWL and SLPD (Table 5). It is also possible to observe two distinct clusters of points in the phylogenetic regression analyses between the scores of the first climatic component derived from the geographical points of species occurrence and the fitted values of both SLPD and RWU (Fig 3). These two clouds correspond to species from which individuals were collected in localities from the Atlantic Forest and the Cerrado, respectively. Species collected at sites in the Atlantic Forest and Cerrado did not differ in REWL (F = 1.092, *p* = 0.24) and RWU (F = 0.156, *p* = 0.65). Otherwise, species collected at the Cerrado sites showed significantly higher SLPD than species collected at the Atlantic Forest sites (F = 3.636, *p* = 0.05).

**Table 5:** Results of phylogenetic regression analyses testing the effects of climatic components on anuran physiological variables corrected by size.

Variable	Phylogenetic signal	Factor	Slope	*P*
		Intercept	-0.002	0.684
**SLPD**	λ = 0.124	C1	0.01	**0.043**
		C2	0.006	0.193
		Intercept	0.01	0.848
**REWL**	λ = 0.175	C1	-0.045	0.361
		C2	-0.018	0.717
		Intercept	-0.002	0.984
**RWU**	λ = 1.119	C1	0.054	**0.001**
		C2	-0.035	0.308

SLPD: sensitivity of locomotor performance to dehydration, considered as the hydration state that results in 70% of maximum performance; REWL: resistance to evaporative water loss; RWU: rates of water uptake; C1: component 1; C2: component 2. Significant probabilities (*P* ≤ 0.05) are highlighted in boldface.

## Discussion

Our interspecific comparative analysis showed that some physiological traits of water balance in anurans, particularly REWL and RWU, show pervasive allometric relations with body mass. Additionally, the analyses showed an association of interspecific variation in RWU and SLPD with climatic characteristics associated with geographical distribution. These results, based on phylogenetically informed analyses, suggest a pattern of adaptation of anuran water balance to abiotic conditions.

REWL declined with an increase in body mass whereas RWU increased with body mass. The relationship between REWL and body mass is likely a consequence of surface area/volume ratio [9,26,38], providing smaller anurans a higher water loss surface. Indeed, the higher tolerance to evaporative water loss of smaller animals has been interpreted as an adaptation associated with this surface area/volume ratio disadvantage [39]. Our results suggest that a higher REWL might at least partially compensate for the increased rates of water loss associated with the evolution of smaller body masses in anurans. These results differ from those of previous studies that did not find a relation between interspecific variation in anuran REWL and body mass [10,40]. However, these previous studies included species characterized by very high REWL and relatively low body mass, and these species might, at least in part, mask the allometric function of REWL. A positive allometric association of RWU with body mass has also been previously suggested to exist to some extent in anurans [19–21]. According to these authors, the required time to reach maximum blood cell flux through the pelvic patch increases with body mass. Otherwise, the magnitude of this flux seems to be related to the environment, given that species from xeric environments show higher flux than species from mesic environments, despite the differences in body mass [19–21]. Our results corroborate the previous positive correlation between RWU and body mass and are based on a phylogenetically controlled analysis that includes a larger number of species. This association of RWU with body mass might be functionally related to the fact that anurans characterized by higher body masses show lower surface area/volume ratio, and it might be associated with a lower pelvic patch area. However, large anurans still need absolutely more water to rehydrate. In this way, our results suggest that a higher hydration rate might be selected to compensate for increased body size. The underlying mechanisms of the body size-related differences in RWU still remain to be investigated, but they might be associated with differences in vascularization [18] and permeability of the pelvic patch due to the density of aquaporins [41–43].

We also found a clear pattern of association between interspecific variation in RWU and SLPD with climatic variables extracted from the geographical points of occurrence for the different species included in this analysis. In particular, the species with geographical occurrence encompassing areas characterized by higher and more seasonally uniform temperatures, and lower and more seasonally concentrated precipitation, had higher RWU and SLPD. Moreover, species collected at sites within the domains of the Atlantic Forest and the Cerrado form two dissociated clusters of data distribution in the phylogenetic regression analyses between these physiological variables and the first climatic component derived from the geographical points of occurrence, reinforcing the pattern that emerged from the continuous covariation. Although these data clearly show the association between the phenotypic variables and the climate of the occurrence points, these data do not allow verification of the contribution of different processes underlying this phenotypic variation (genetic adaptation or acclimatization). Phenotypic plasticity might even play a significant role determining the interspecific physiological variation associated with seasonal acclimatization [10,11], given that individuals from different species were collected and measured at different seasons. The analysis of populations from the same species collected in both biomes, as well as the investigation of the acclimation capacity of these physiological variables, might shed light on these topics. The inclusion of species inhabiting environments characterized by more drastic water restriction, such as arid and semi-arid localities, might also expand the knowledge of patterns of water balance adaptations in anurans.

Several authors have previously reported that terrestrial or arid-dwelling species of anurans show higher hydration rates when compared to semi-aquatic species or to those occurring in mesic environments [17,20,21]. These results are consistent with morphological observations showing that species from xeric environments have more vascularized ventral skin [18]. These joint results suggest a pattern of directional selection of individuals able to hydrate more efficiently and at faster rates once they find water sources in environments where water represents a scarce resource. Again, our results support these previous results and interpretations through a comparative and phylogenetically informed approach. It is important to highlight that the interspecific variation in RWU reported here was based on measurements from a free water surface, a situation that might not be ecologically relevant for many species. In the field, many species might actually rehydrate more frequently from humid soil, mainly outside of the breeding season [44]. In this way, RWU from a free water surface might not be directly selected in nature, but it might be functionally associated with differences in efficiency of rehydration from humid substrates. This hypothesis remains to be tested.

Previous comparative studies have found a pattern of negative association between SLPD and the occupation of open and more water-restricted environments [6–8]. These previous results suggest that individuals who are able to maintain behavioral performance at lower states of hydration are under directional selection in water-restricted environments. Our present analysis showed the opposite pattern: species with geographical distribution more tightly associated with mesic environments show lower SLPD than species with geographical distribution encompassing more water-restricted environments. Although our results contradict previous studies, we believe that this opposite pattern to those described for previous studies might be associated with underlying methodological differences. The present study was conducted with a much higher number of species, split into several phylogenetic lineages, and incorporated phylogenetically corrected analyses. Furthermore, our study analyzed the association between the physiological traits and climatic data instead of collecting data exclusively on physiology and associating it to namely characterized environments. In this way, we believe that our results are robust and that this pattern might be associated with selection on temporal patterns of activity in these different environments. In environments characterized by lower and more seasonally concentrated precipitation, such as the Cerrado, individuals might be selected to concentrate reproduction and other general activities, such as foraging, to restricted periods with a higher probability of precipitation. This concentrated pattern of activity might prevent selection from acting on the sensitivity of locomotion to dehydration in the Cerrado. Otherwise, species from environments characterized by higher and seasonally distributed precipitation might maintain continuous activity, with several species showing year-round reproduction or at least foraging activity [45–49]. This sustained activity at periods of lower relative humidity might allow directional selection on SLPD in the Atlantic Forest. This reasoning remains largely speculative, and comparative studies including the duration of reproductive season on different localities are necessary to test hypotheses along these lines. Previous studies [6–8] also may not be comparable to the present one because they tested SLPD at different temperatures and showed that patterns of adaptation and/or acclimatization can shift the curves, resulting in a reduction in the SLPD at temperatures closer to the temperatures of activity for different species in the field. In the present study, we performed tests of SLPD at a single temperature. In this way, we need to consider that species from the Atlantic Forest and Cerrado might show different optimum temperatures where the effects of dehydration are reduced, and the comparative analysis of SLPD at these specific temperatures might change the patterns described here.

Our analysis did not recover an interspecific covariation between REWL and the climatic variables associated with geographical distribution, corroborating results from previous studies that attempted to associate variation in REWL with differences in habitat [38,50–54]. These joint results do not corroborate the long-lasting corollary reasoning that high skin permeability would be a direct limitation for the occupation of more water-restricted environments by amphibians and that individuals displaying high REWL and inhabiting these environments would be strongly selected. It is possible that behavioral adjustments, such as on patterns of time of reproduction and microenvironmental selection during activity might also prevent directional selection on REWL. Furthermore, several studies have emphasized a consistent pattern of anuran interspecific association between REWL and the habit, with arboreal species displaying higher values than terrestrial and semi-aquatic ones [9,10,55]. Although we sampled a relatively high number of species for the present investigation, the interspecific variation in habits are highly skewed through the phylogeny, preventing the analysis of the relationship between REWL and habits in this study.

In summary, this comparative analysis of anuran water balance showed that body mass coevolved with REWL and RWU in opposite allometric directions. In this way, species with higher body mass show lower REWL and higher RWU than species with lower body mass. Additionally, species inhabiting areas characterized by higher and more seasonally uniform temperatures, and lower and more seasonally concentrated precipitation, had higher RWU and SLPD than species with geographical distributions that were more restricted to mesic environments. These results suggest that the ability to hydrate faster from a free water surface might indicate an adaptation of anurans to environments characterized by a higher seasonal restriction on water availability. These differences in RWU from a free water surface might be, alternatively, associated with efficiency of water uptake from humid substrates. Otherwise, the higher SLPD displayed by anurans inhabiting the Cerrado might be related to a more intense restriction of activity at the peak of the rainy season, precluding the action of selection on this variable.

## Supporting Information

S1 Fig Geographical distribution of *Dendropsophus microps*. Collection site of individuals for physiological measures, points of occurrence for the species [29] and areas of Atlantic Forrest and Cerrado domains [23].
(TIF)

S2 Fig Geographical distribution of *Dendropsophus minutus*. Collection site of individuals for physiological measures, points of occurrence for the species [29] and areas of Atlantic Forrest and Cerrado domains [23].
(TIF)

S3 Fig Geographical distribution of *Scinax rizibilis*. Collection site of individuals for physiological measures, points of occurrence for the species [29] and areas of Atlantic Forrest and Cerrado domains [23].
(TIF)

S4 Fig Geographical distribution of *Scinax crospedospilus*. Collection site of individuals for physiological measures, points of occurrence for the species [29] and areas of Atlantic Forrest and Cerrado domains [23].
(TIF)

S5 Fig Geographical distribution of *Scinax hayii*. Collection site of individuals for physiological measures, points of occurrence for the species [29] and areas of Atlantic Forrest and Cerrado domains [23].
(TIF)

S6 Fig Geographical distribution of *Hypsiboas albopuctatus*. Collection site of individuals for physiological measures, points of occurrence for the species [29] and areas of Atlantic Forrest and Cerrado domains [23].
(TIF)

S7 Fig Geographical distribution of *Hypsiboas faber*. Collection site of individuals for physiological measures, points of occurrence for the species [29] and areas of Atlantic Forrest and Cerrado domains [23].
(TIF)

S8 Fig Geographical distribution of *Hypsiboas bischoffi*. Collection site of individuals for physiological measures, points of occurrence for the species [29] and areas of Atlantic Forrest and Cerrado domains [23].
(TIF)

S9 Fig Geographical distribution of *Hypsiboas polytaenius*. Collection site of individuals for physiological measures, points of occurrence for the species [29] and areas of Atlantic Forrest and Cerrado domains [23].
(TIF)

S10 Fig Geographical distribution of *Proceratophrys boiei*. Collection site of individuals for physiological measures, points of occurrence for the species [29] and areas of Atlantic Forrest and Cerrado domains [23].
(TIF)

S11 Fig Geographical distribution of *Leptodactylus notoaktites*. Collection site of individuals for physiological measures, points of occurrence for the species [29] and areas of Atlantic Forrest and Cerrado domains [23].
(TIF)

S12 Fig Geographical distribution of *Leptodactylus podicipinus*. Collection site of individuals for physiological measures, points of occurrence for the species [29] and areas of Atlantic Forrest and Cerrado domains [23].
(TIF)

S13 Fig Geographical distribution of *Physalaemus olfersii*. Collection site of individuals for physiological measures, points of occurrence for the species [29] and areas of Atlantic Forrest and Cerrado domains [23].
(TIF)

S14 Fig Geographical distribution of *Physalaemus spiniger*. Collection site of individuals for physiological measures, points of occurrence for the species [29] and areas of Atlantic Forrest and Cerrado domains [23].
(TIF)

S15 Fig Geographical distribution of *Rhinella ornata*. Collection site of individuals for physiological measures, points of occurrence for the species [29] and areas of Atlantic Forrest and Cerrado domains [23].
(TIF)

S16 Fig Geographical distribution of *Rhinella icterica*. Collection site of individuals for physiological measures, points of occurrence for the species [29] and areas of Atlantic Forrest and Cerrado domains [23].
(TIF)

S17 Fig Geographical distribution of *Rhinella schneideri*. Collection site of individuals for physiological measures, points of occurrence for the species [29] and areas of Atlantic Forrest and Cerrado domains [23].
(TIF)

S18 Fig Locomotor performance of *Dendropsophus microps*. Mean locomotor performance transformed as a percentage of maximum performance in different hydration levels.
(TIF)

S19 Fig Locomotor performance of *Dendropsophus minutus*. Mean locomotor performance transformed as a percentage of maximum performance in different hydration levels.
(TIF)

S20 Fig Locomotor performance of *Scinax hayii*. Mean locomotor performance transformed as a percentage of maximum performance in different hydration levels.
(TIF)

S21 Fig Locomotor performance of *Hypsiboas albopuctatus*. Mean locomotor performance transformed as a percentage of maximum performance in different hydration levels.
(TIF)

S22 Fig Locomotor performance of *Hypsiboas faber*. Mean locomotor performance transformed as a percentage of maximum performance in different hydration levels.
(TIF)

S23 Fig Locomotor performance of *Hypsiboas bischoffi*. Mean locomotor performance transformed as a percentage of maximum performance in different hydration levels.
(TIF)

S24 Fig Locomotor performance of *Hypsiboas polytaenius*. Mean locomotor performance transformed as a percentage of maximum performance in different hydration levels.
(TIF)

S25 Fig Locomotor performance of *Proceratophrys boiei*. Mean locomotor performance transformed as a percentage of maximum performance in different hydration levels.
(TIF)

S26 Fig Locomotor performance of *Leptodactylus podicipinus*. Mean locomotor performance transformed as a percentage of maximum performance in different hydration levels.
(TIF)

S27 Fig Locomotor performance of *Physalaemus olfersii*. Mean locomotor performance transformed as a percentage of maximum performance in different hydration levels.
(TIF)

S28 Fig Locomotor performance of *Rhinella ornata*. Mean locomotor performance transformed as a percentage of maximum performance in different hydration levels.
(TIF)

S29 Fig Locomotor performance of *Rhinella icterica*. Mean locomotor performance transformed as a percentage of maximum performance in different hydration levels.
(TIF)

S30 Fig Locomotor performance of *Rhinella schneideri*. Mean locomotor performance transformed as a percentage of maximum performance in different hydration levels.
(TIF)

S1 Text ARRIVE. Animal Research: Reporting In Vivo Experiments Guidelines Checklist.
(DOCX)
