# Supplementary figures and images for: Relation between Water Balance and Climatic Variables Associated with the Geographical Distribution of Anurans
Source: PLoS One. 2015 Oct 15;10(10):e0140761. doi: 10.1371/journal.pone.0140761 (PMC4607303; doi:10.1371/journal.pone.0140761)

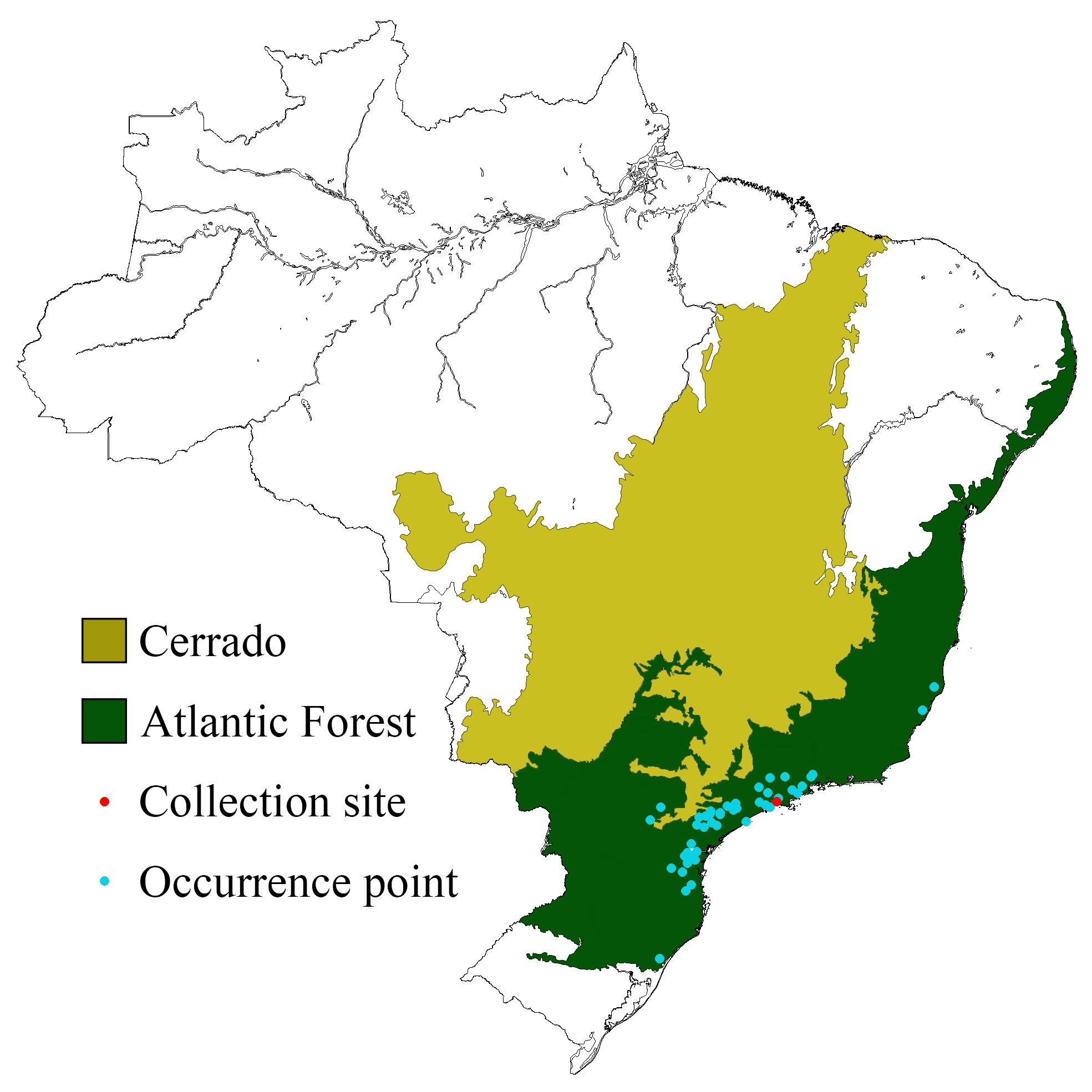

Supplement: S1 Fig — Collection site of individuals for physiological measures, points of occurrence for the species [29] and areas of Atlantic Forrest and Cerrado domains [23]. (TIF) [file pone.0140761.s001.tif]

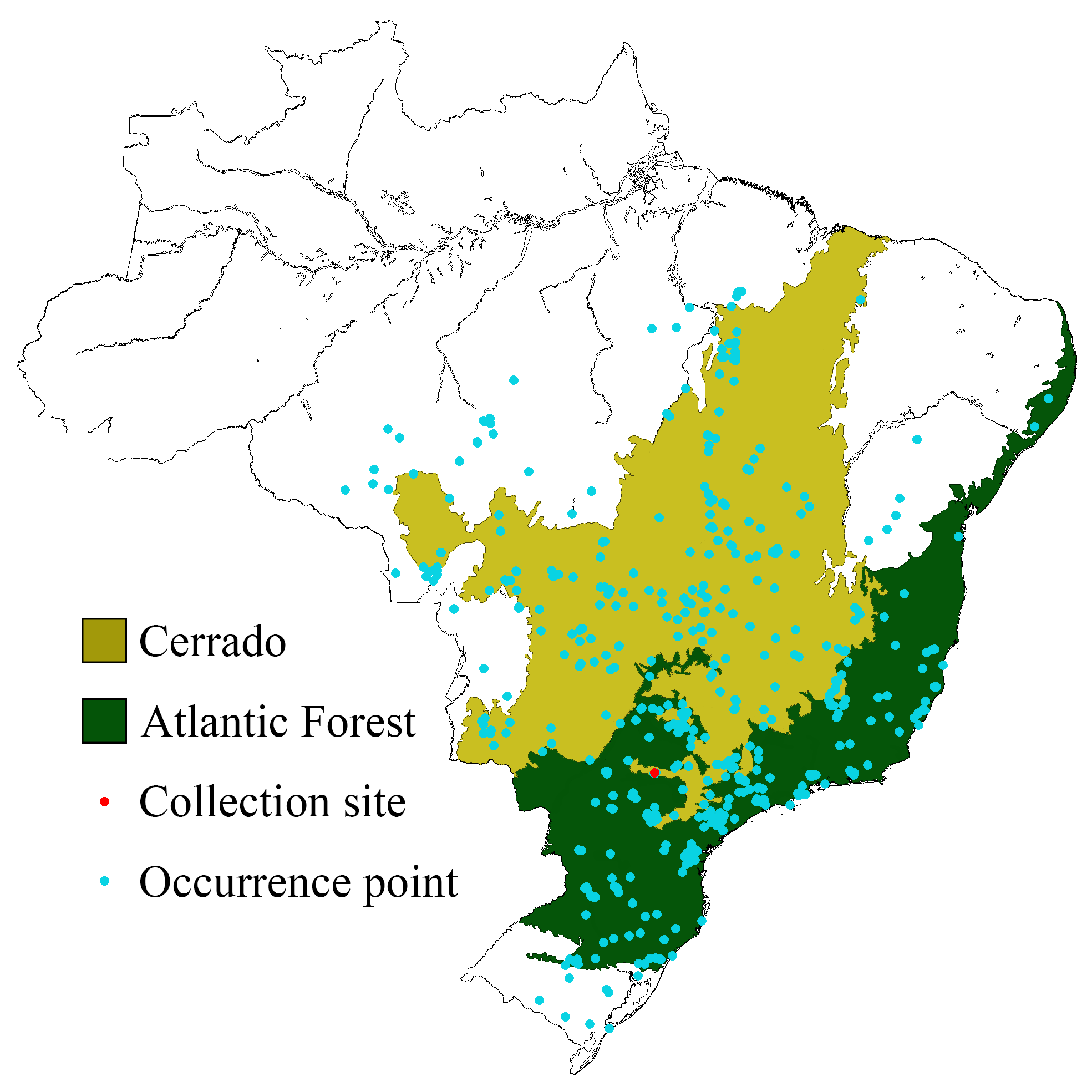

Supplement: S2 Fig — Collection site of individuals for physiological measures, points of occurrence for the species [29] and areas of Atlantic Forrest and Cerrado domains [23]. (TIF) [file pone.0140761.s002.tif]

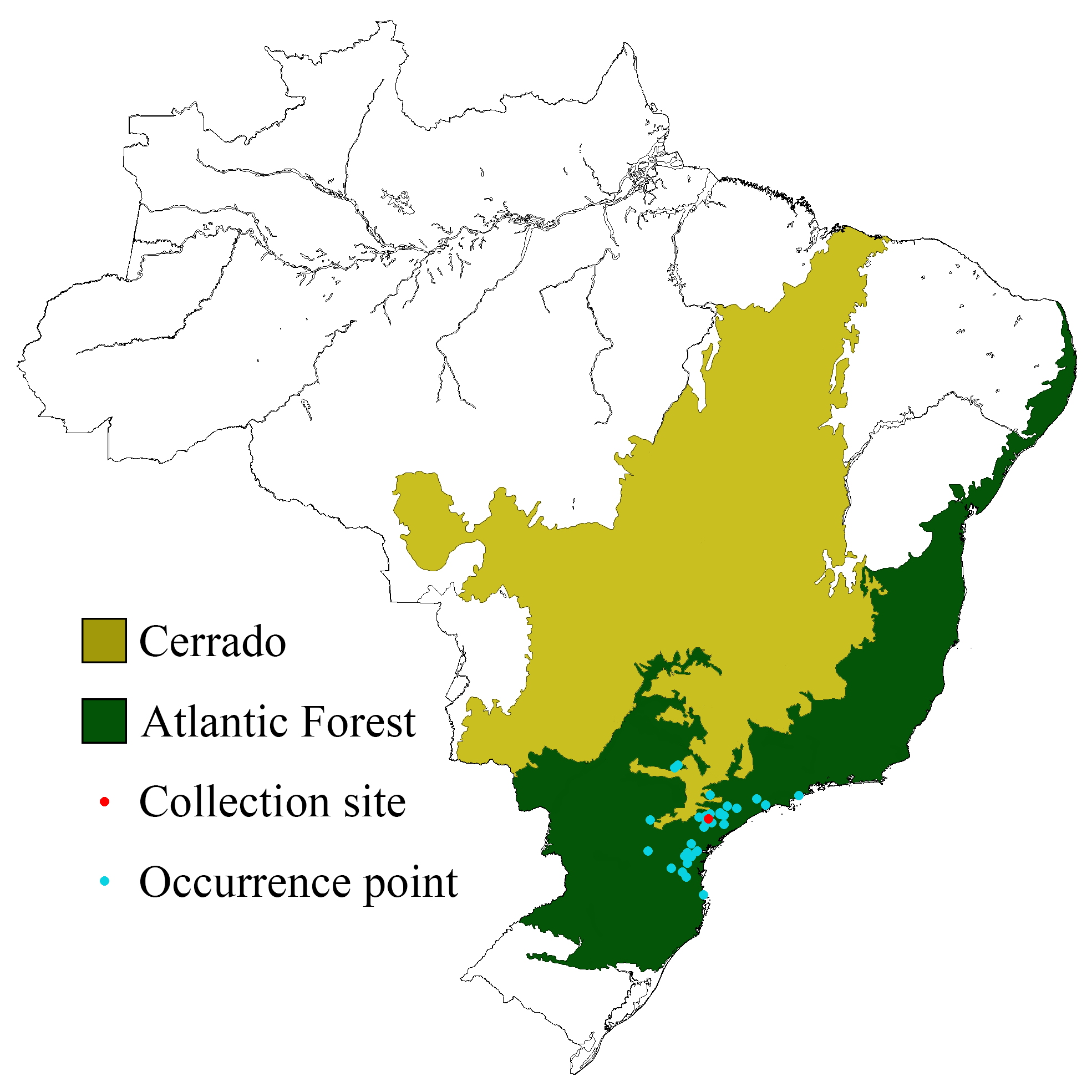

Supplement: S3 Fig — Collection site of individuals for physiological measures, points of occurrence for the species [29] and areas of Atlantic Forrest and Cerrado domains [23]. (TIF) [file pone.0140761.s003.tif]

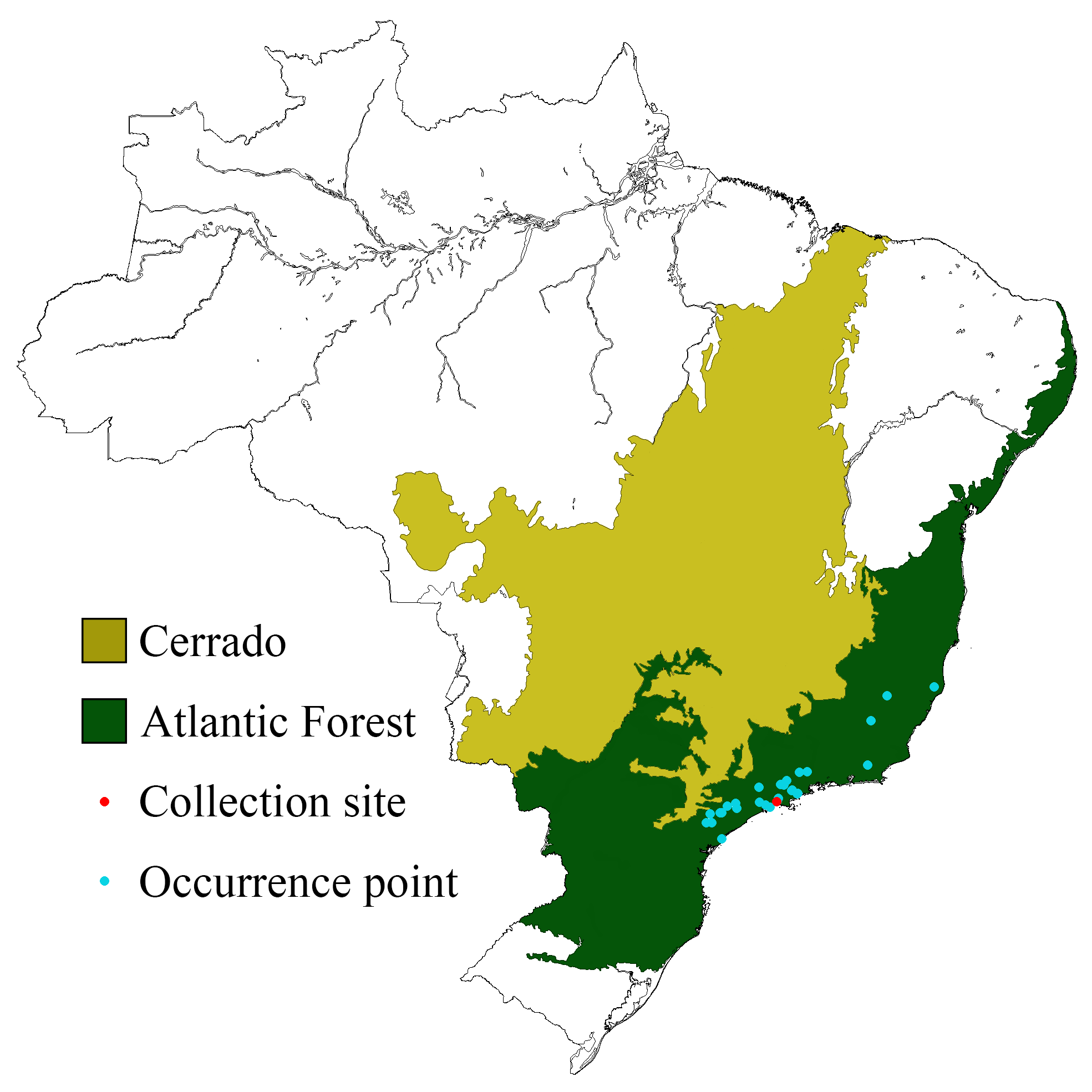

Supplement: S4 Fig — Collection site of individuals for physiological measures, points of occurrence for the species [29] and areas of Atlantic Forrest and Cerrado domains [23]. (TIF) [file pone.0140761.s004.tif]

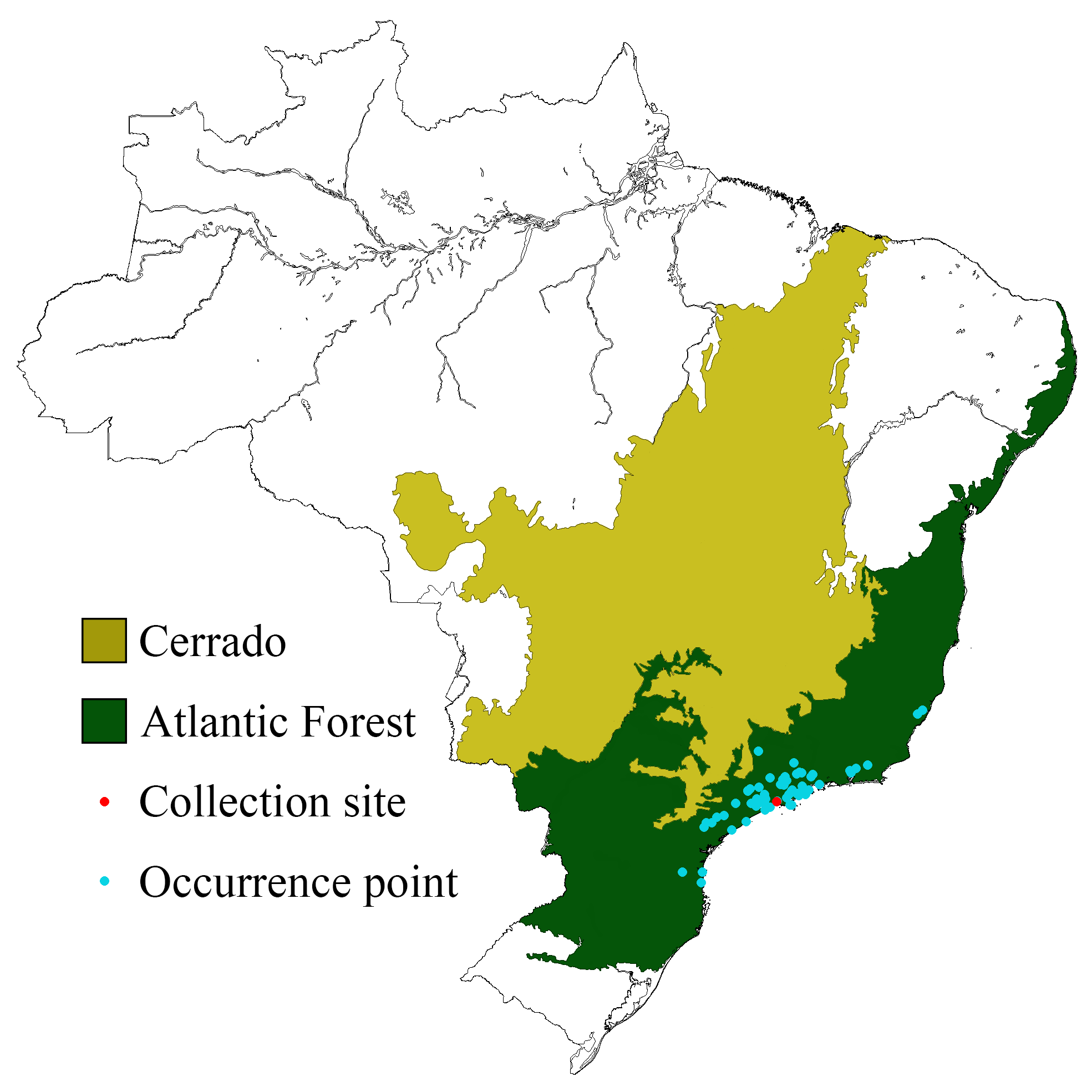

Supplement: S5 Fig — Collection site of individuals for physiological measures, points of occurrence for the species [29] and areas of Atlantic Forrest and Cerrado domains [23]. (TIF) [file pone.0140761.s005.tif]

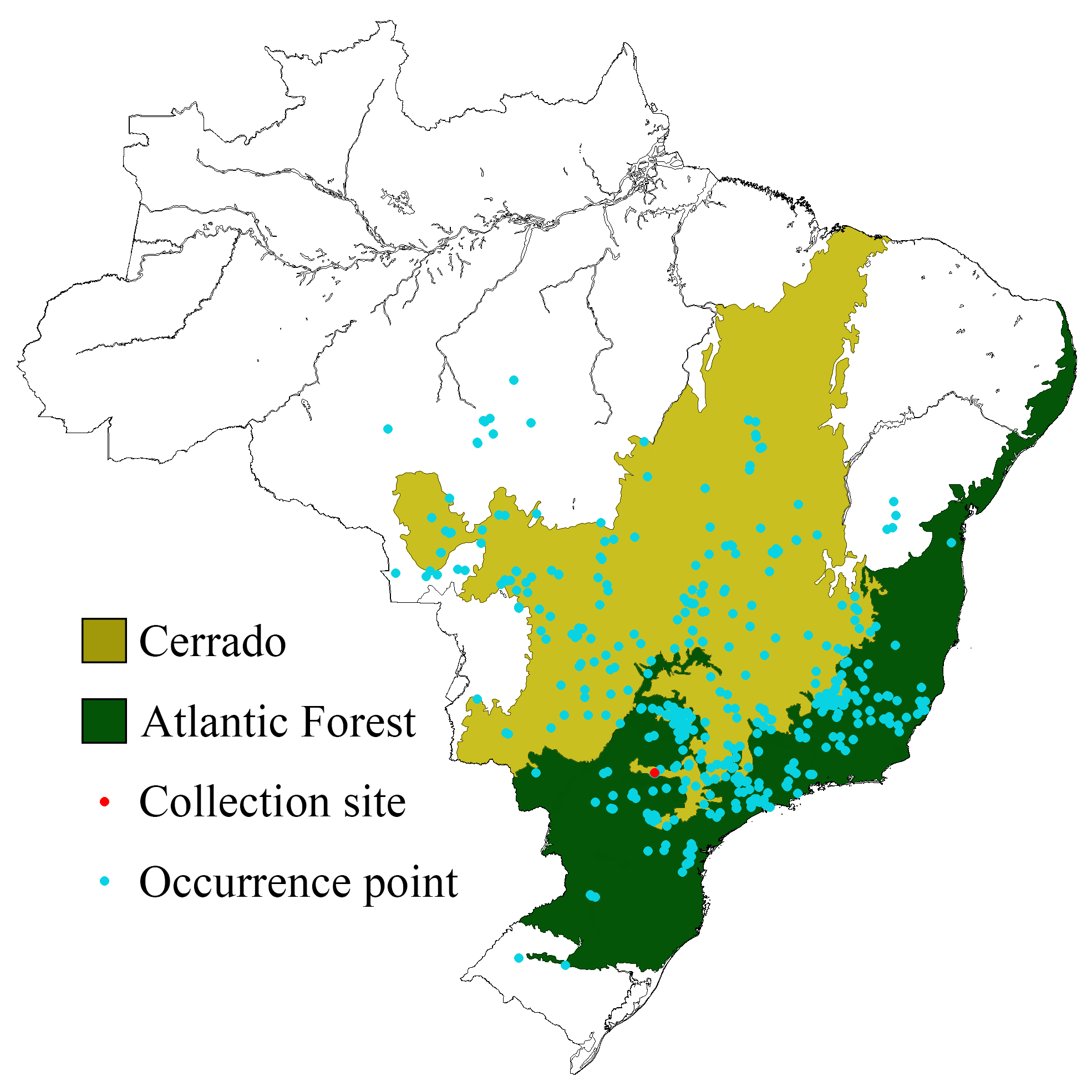

Supplement: S6 Fig — Collection site of individuals for physiological measures, points of occurrence for the species [29] and areas of Atlantic Forrest and Cerrado domains [23]. (TIF) [file pone.0140761.s006.tif]

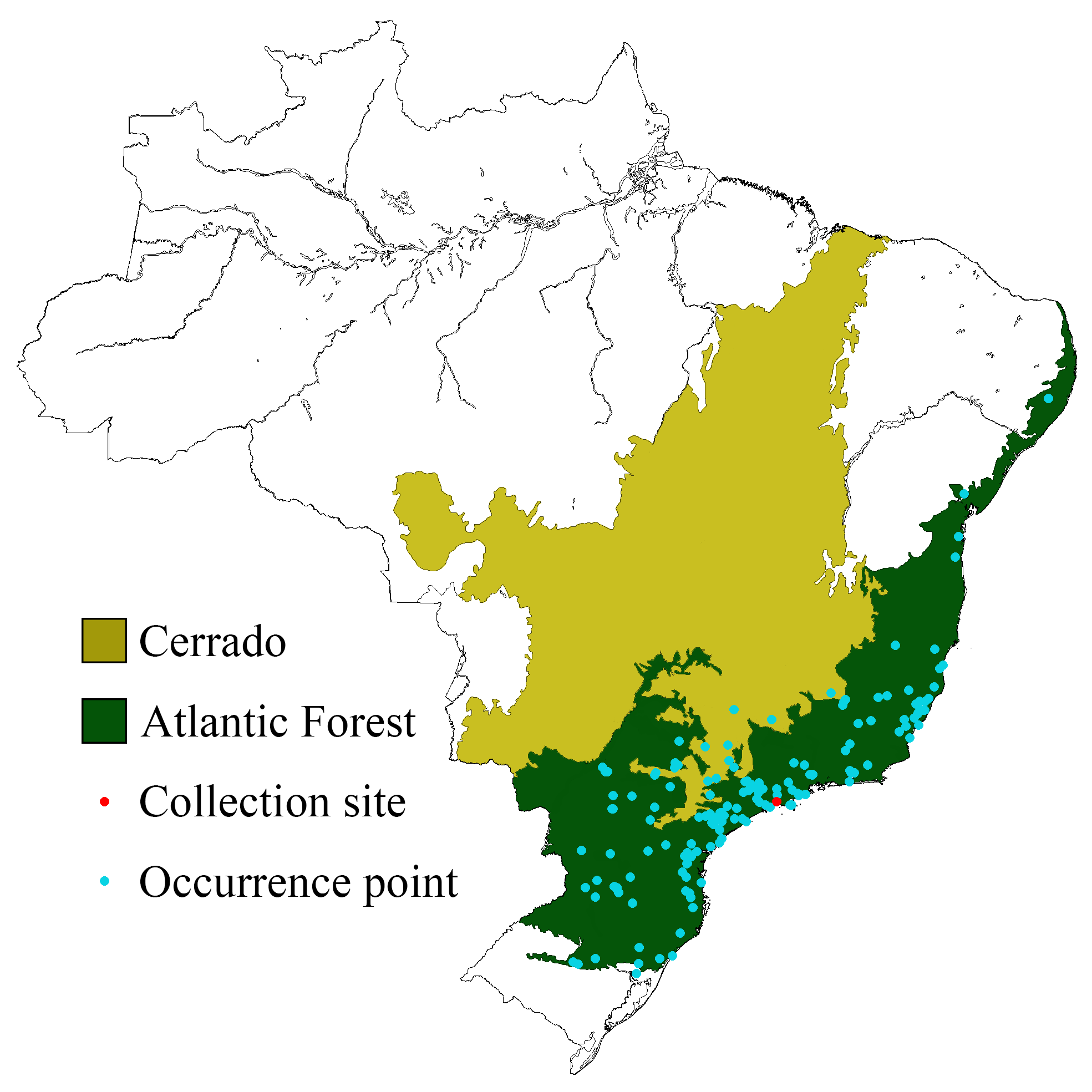

Supplement: S7 Fig — Collection site of individuals for physiological measures, points of occurrence for the species [29] and areas of Atlantic Forrest and Cerrado domains [23]. (TIF) [file pone.0140761.s007.tif]

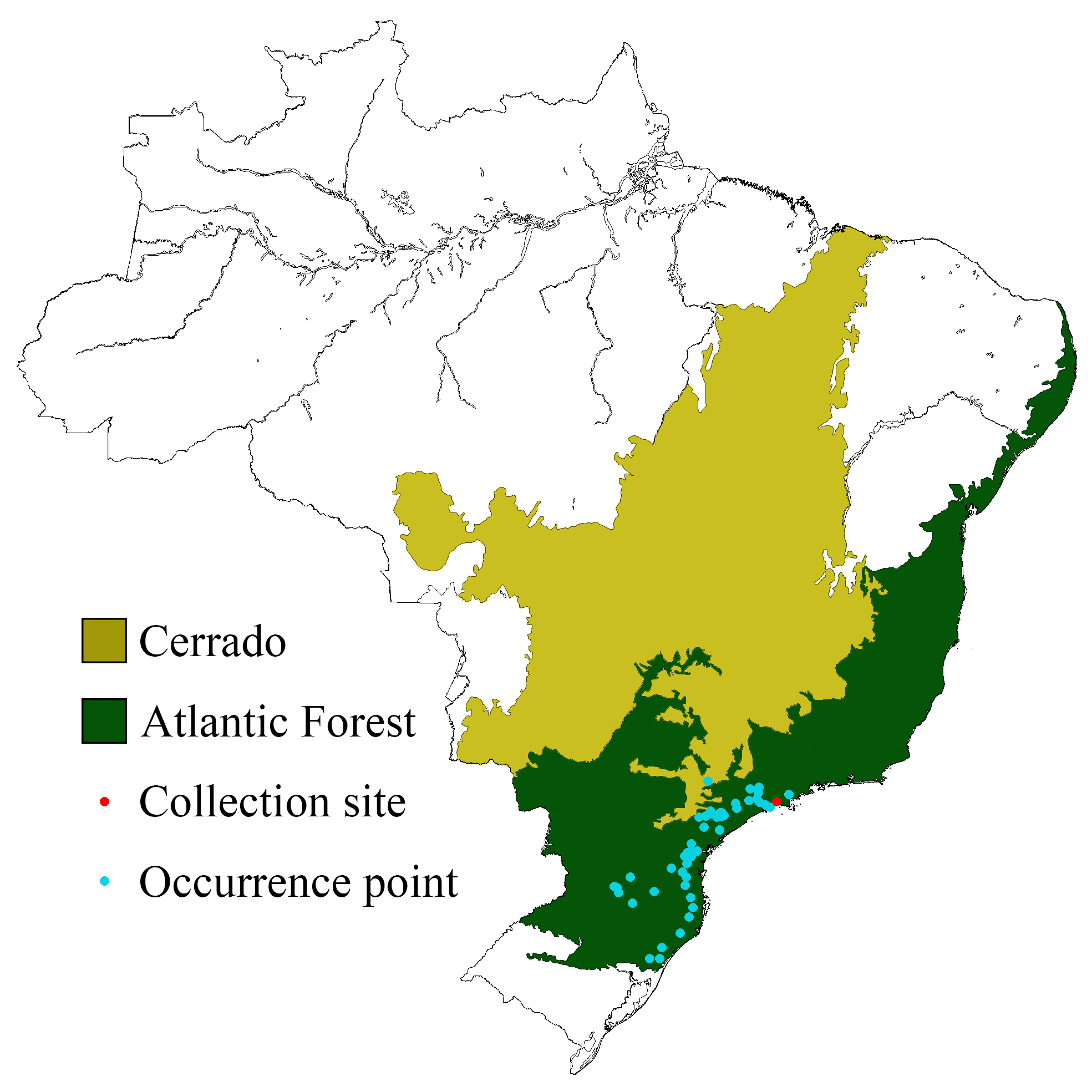

Supplement: S8 Fig — Collection site of individuals for physiological measures, points of occurrence for the species [29] and areas of Atlantic Forrest and Cerrado domains [23]. (TIF) [file pone.0140761.s008.tif]

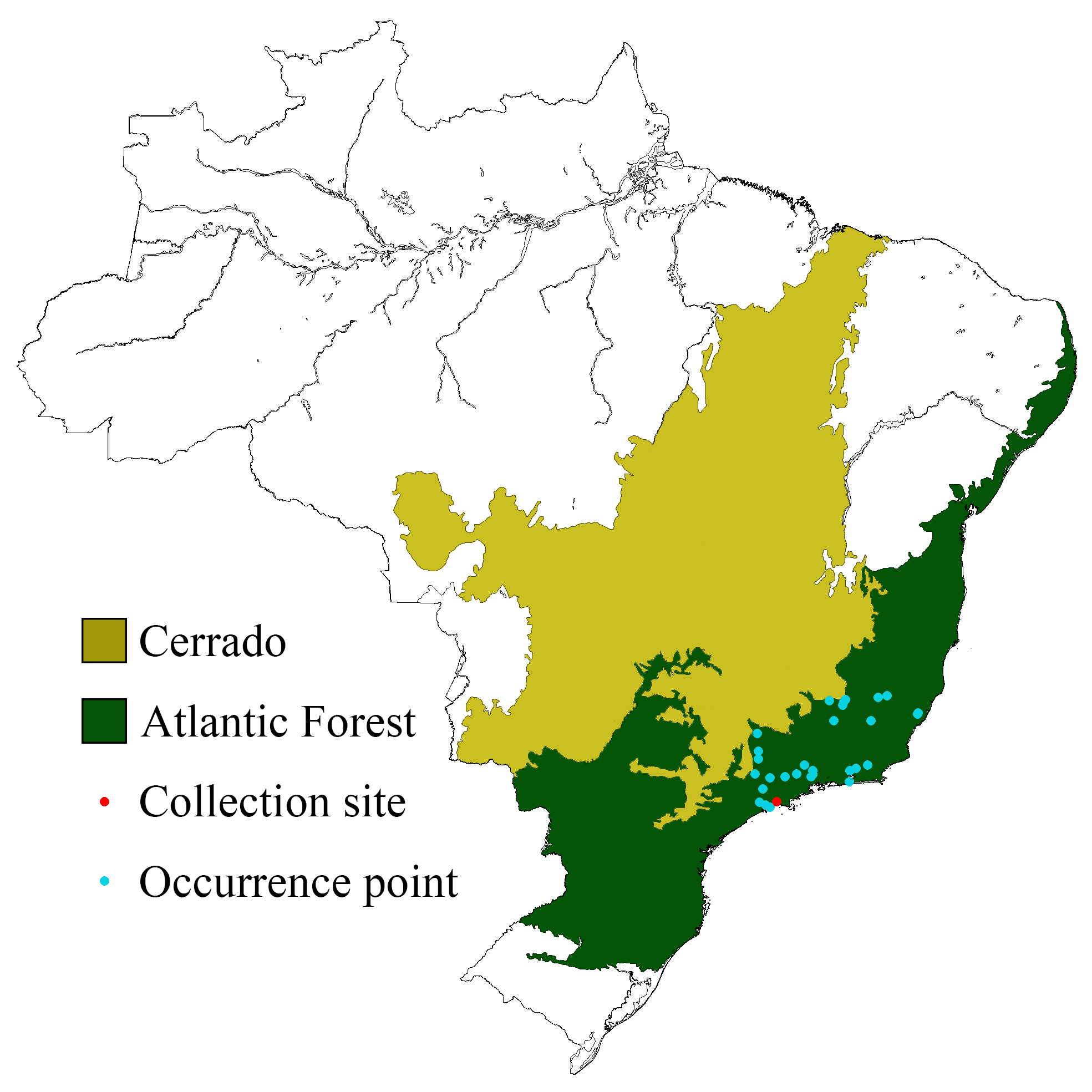

Supplement: S9 Fig — Collection site of individuals for physiological measures, points of occurrence for the species [29] and areas of Atlantic Forrest and Cerrado domains [23]. (TIF) [file pone.0140761.s009.tif]

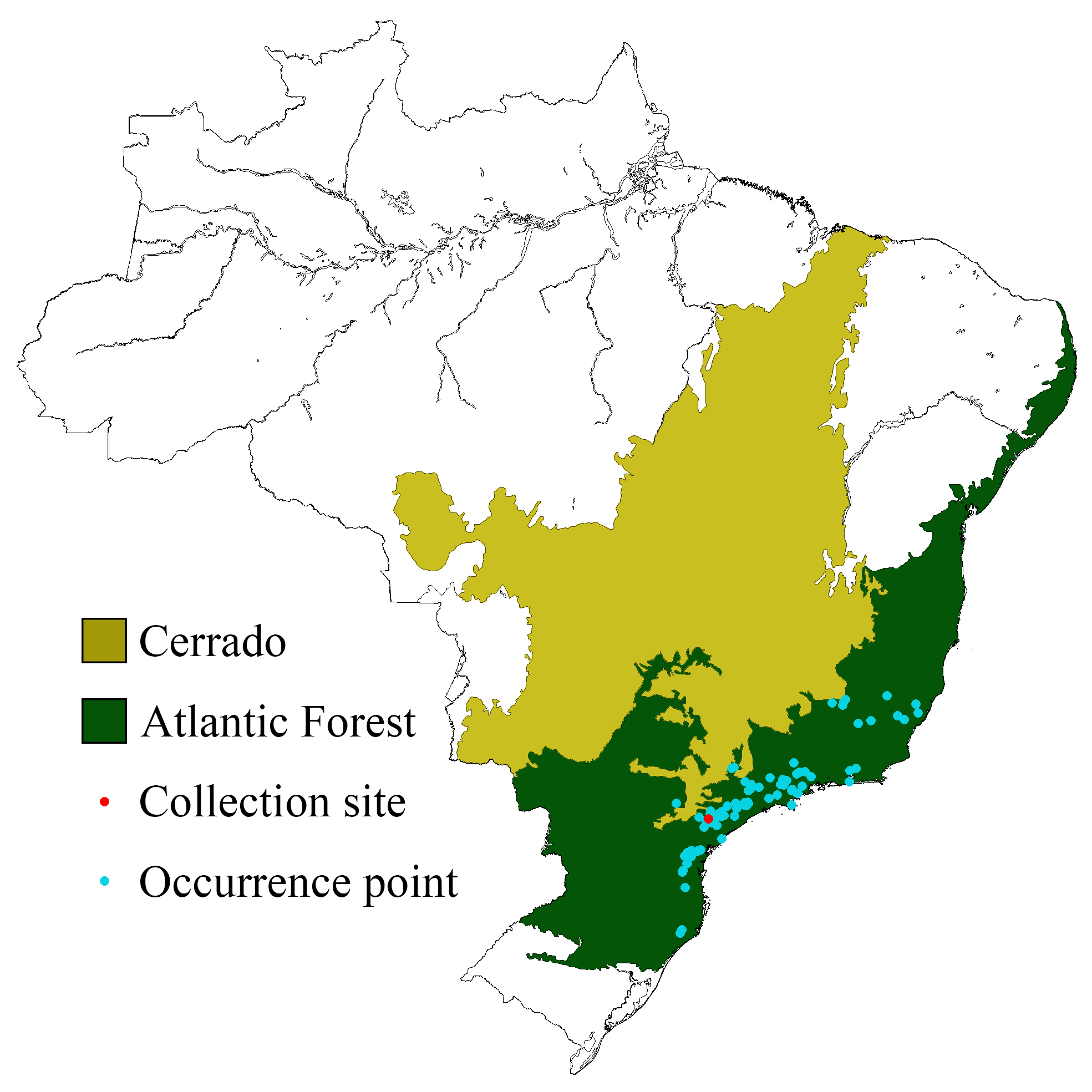

Supplement: S10 Fig — Collection site of individuals for physiological measures, points of occurrence for the species [29] and areas of Atlantic Forrest and Cerrado domains [23]. (TIF) [file pone.0140761.s010.tif]

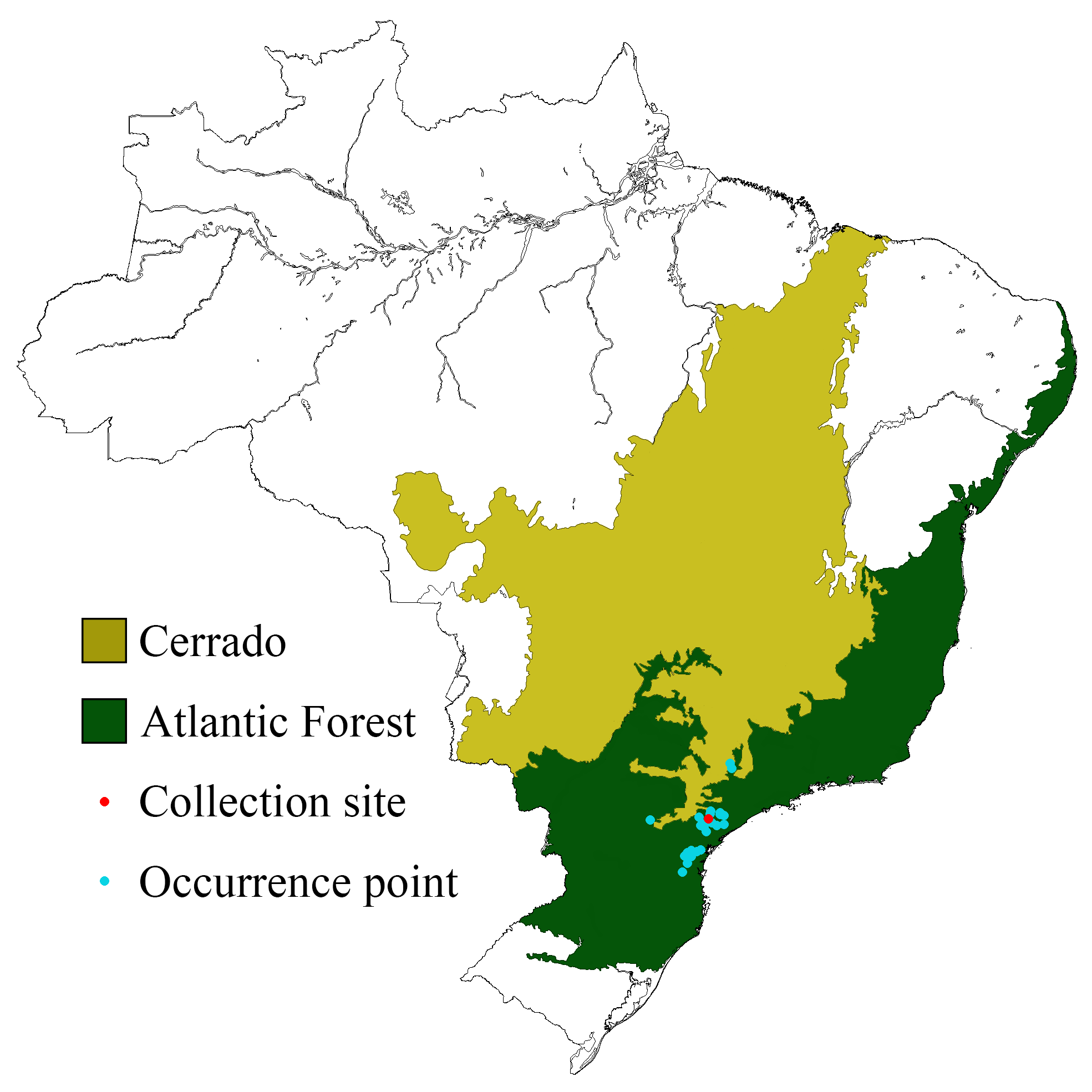

Supplement: S11 Fig — Collection site of individuals for physiological measures, points of occurrence for the species [29] and areas of Atlantic Forrest and Cerrado domains [23]. (TIF) [file pone.0140761.s011.tif]

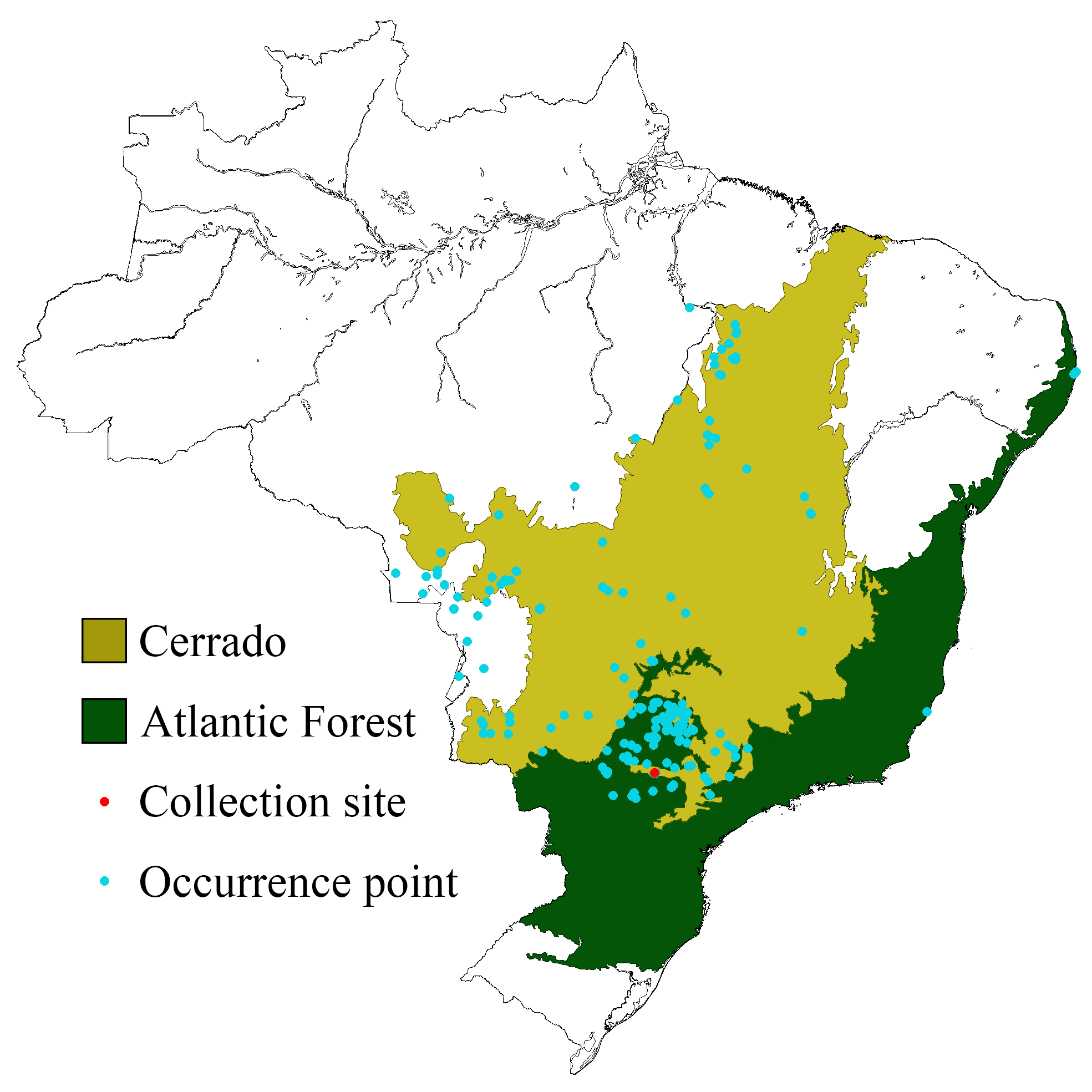

Supplement: S12 Fig — Collection site of individuals for physiological measures, points of occurrence for the species [29] and areas of Atlantic Forrest and Cerrado domains [23]. (TIF) [file pone.0140761.s012.tif]

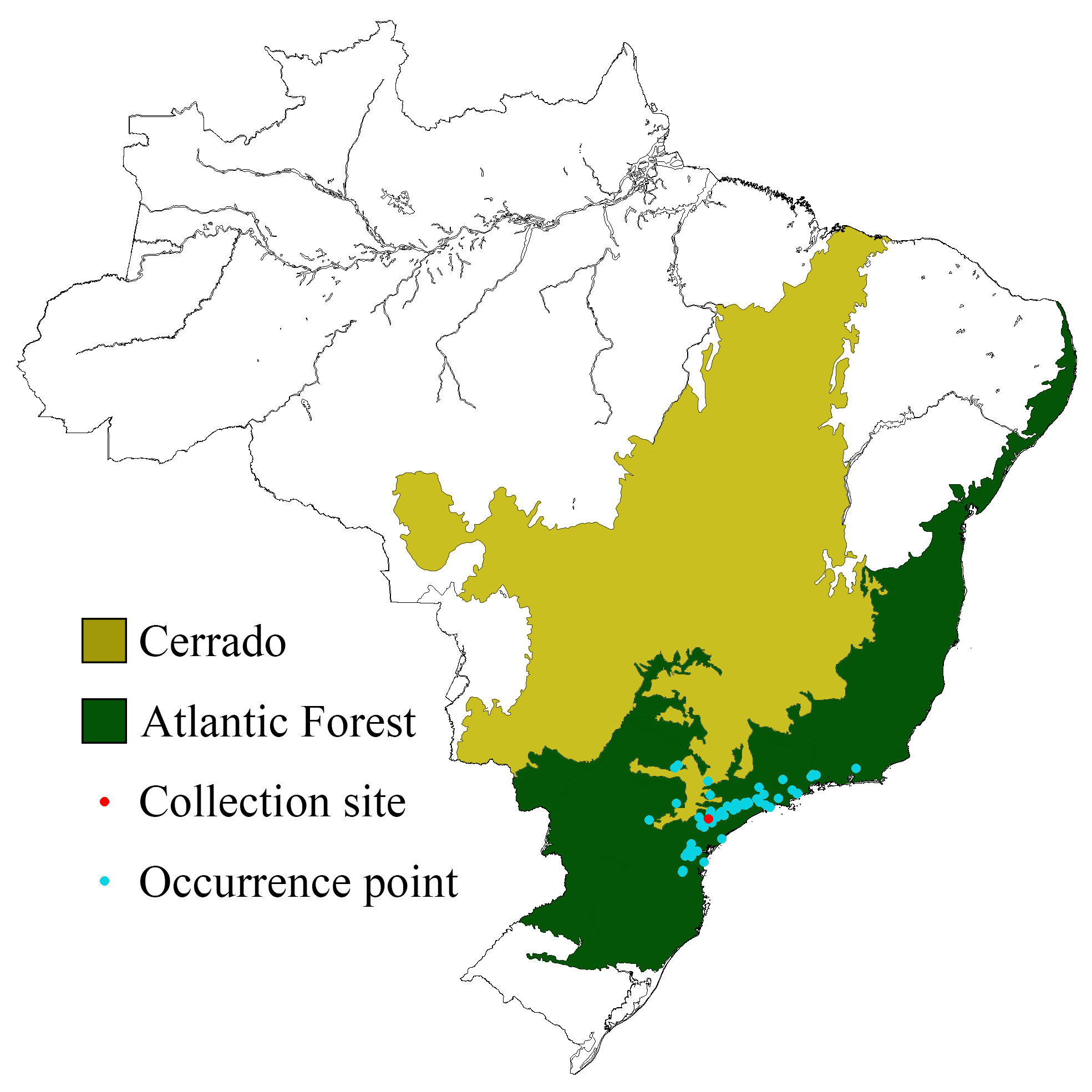

Supplement: S13 Fig — Collection site of individuals for physiological measures, points of occurrence for the species [29] and areas of Atlantic Forrest and Cerrado domains [23]. (TIF) [file pone.0140761.s013.tif]

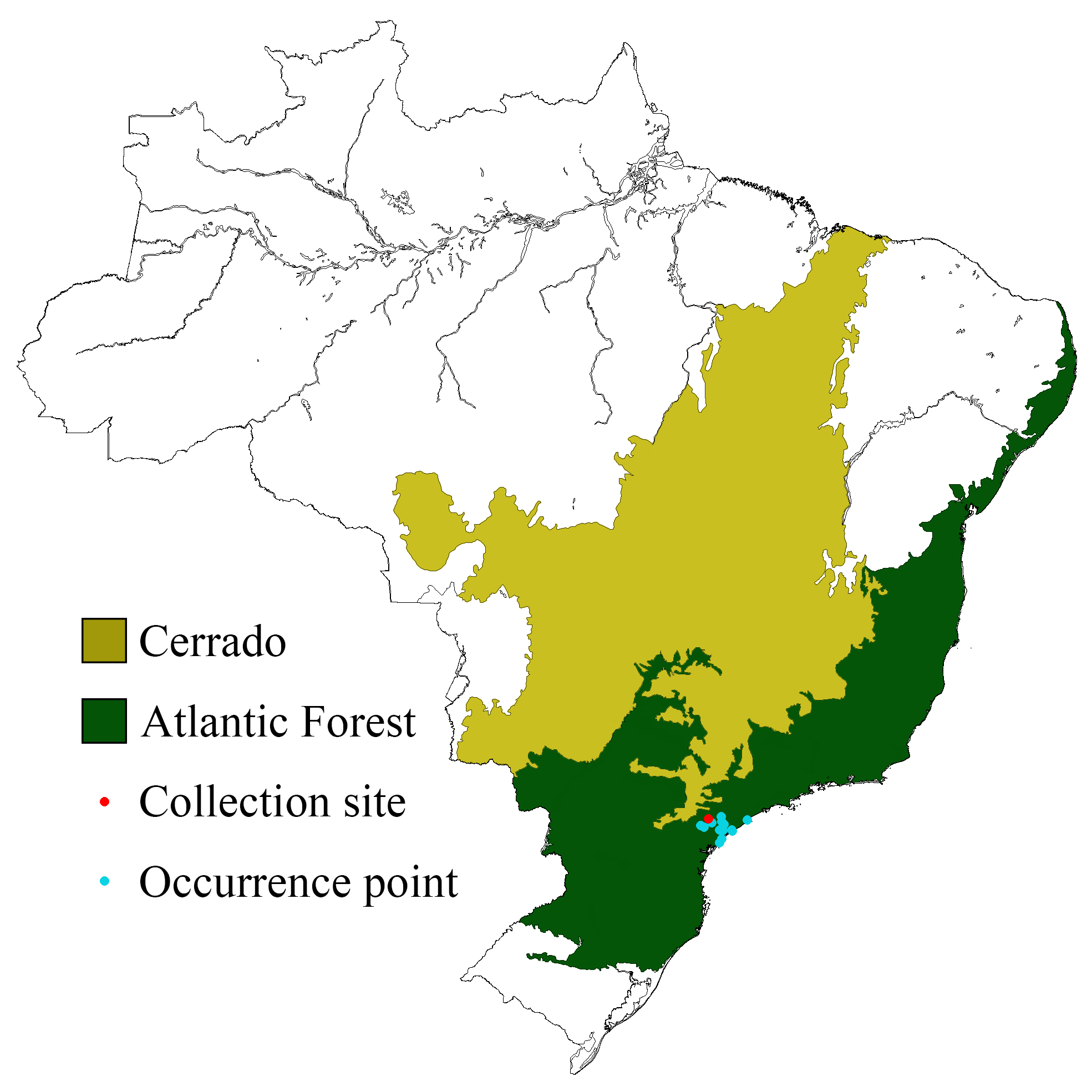

Supplement: S14 Fig — Collection site of individuals for physiological measures, points of occurrence for the species [29] and areas of Atlantic Forrest and Cerrado domains [23]. (TIF) [file pone.0140761.s014.tif]

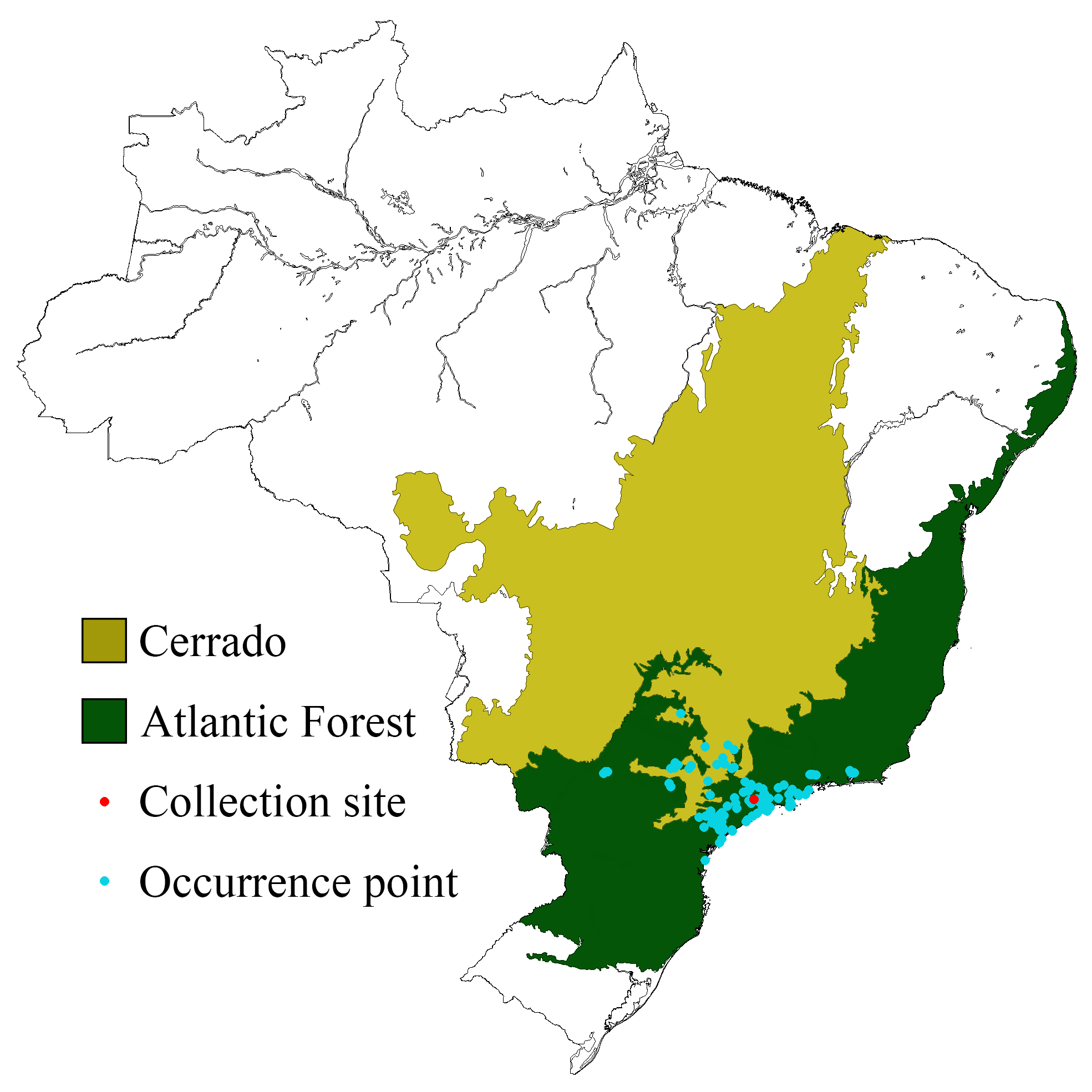

Supplement: S15 Fig — Collection site of individuals for physiological measures, points of occurrence for the species [29] and areas of Atlantic Forrest and Cerrado domains [23]. (TIF) [file pone.0140761.s015.tif]

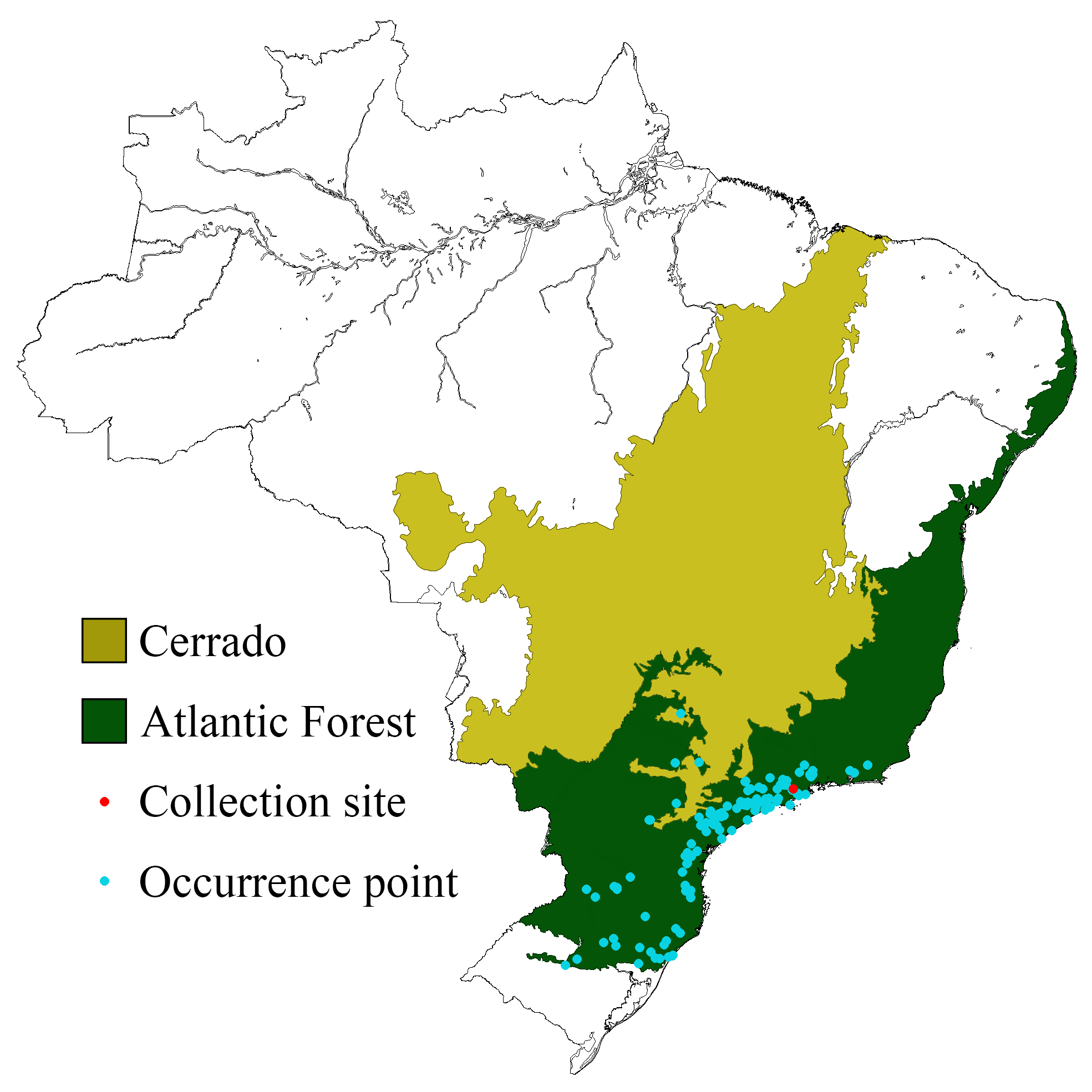

Supplement: S16 Fig — Collection site of individuals for physiological measures, points of occurrence for the species [29] and areas of Atlantic Forrest and Cerrado domains [23]. (TIF) [file pone.0140761.s016.tif]

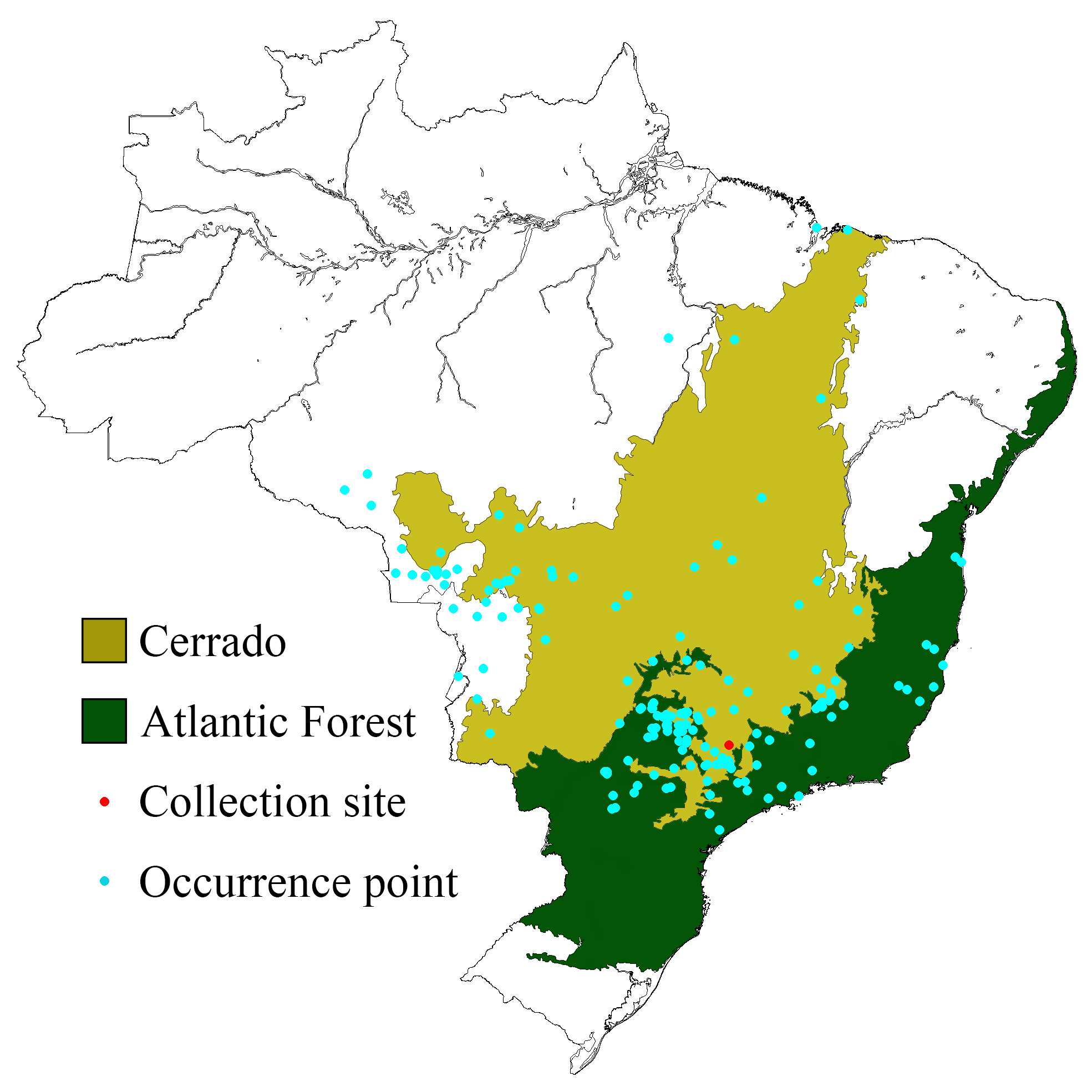

Supplement: S17 Fig — Collection site of individuals for physiological measures, points of occurrence for the species [29] and areas of Atlantic Forrest and Cerrado domains [23]. (TIF) [file pone.0140761.s017.tif]

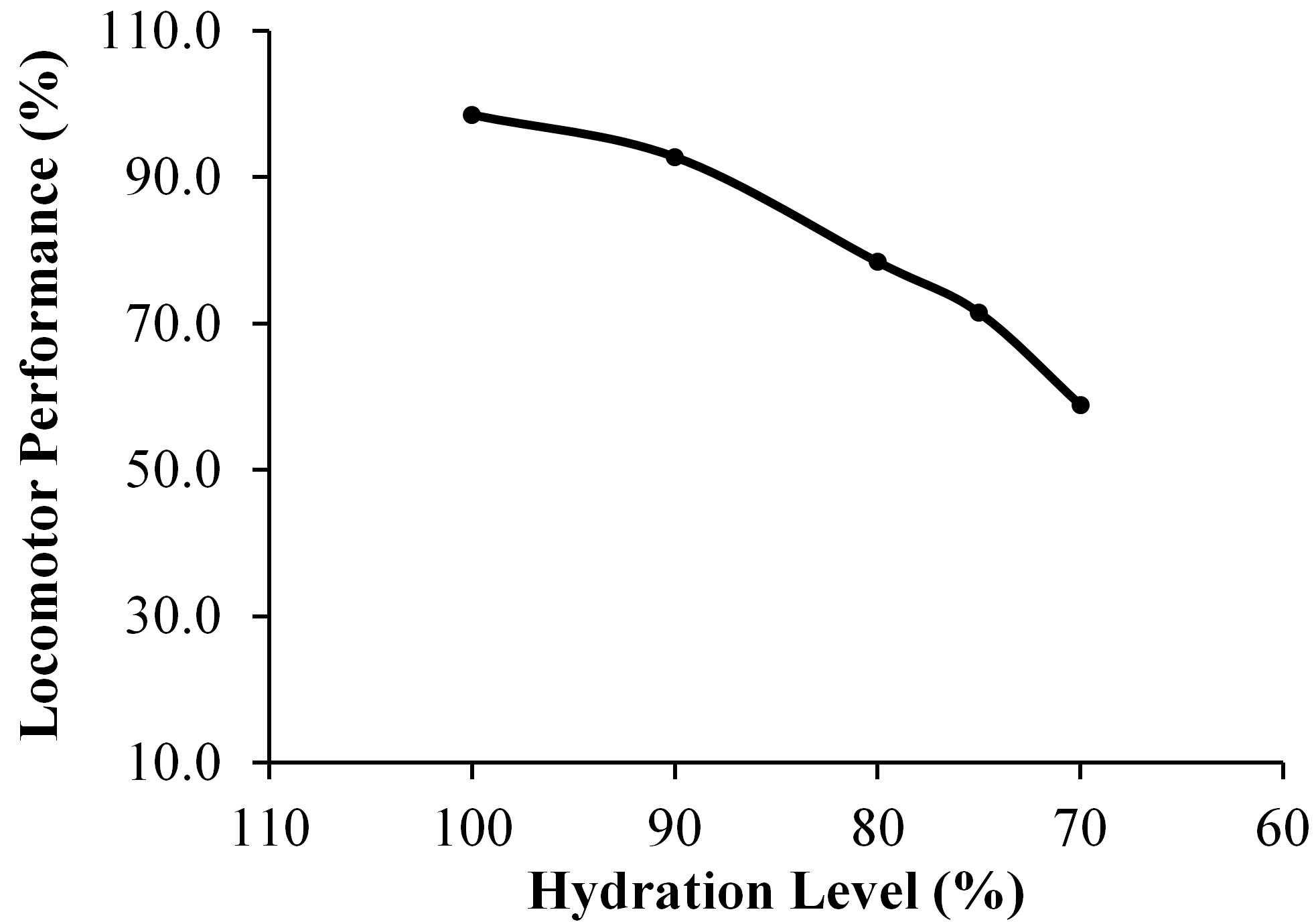

Supplement: S18 Fig — Mean locomotor performance transformed as a percentage of maximum performance in different hydration levels. (TIF) [file pone.0140761.s018.tif]

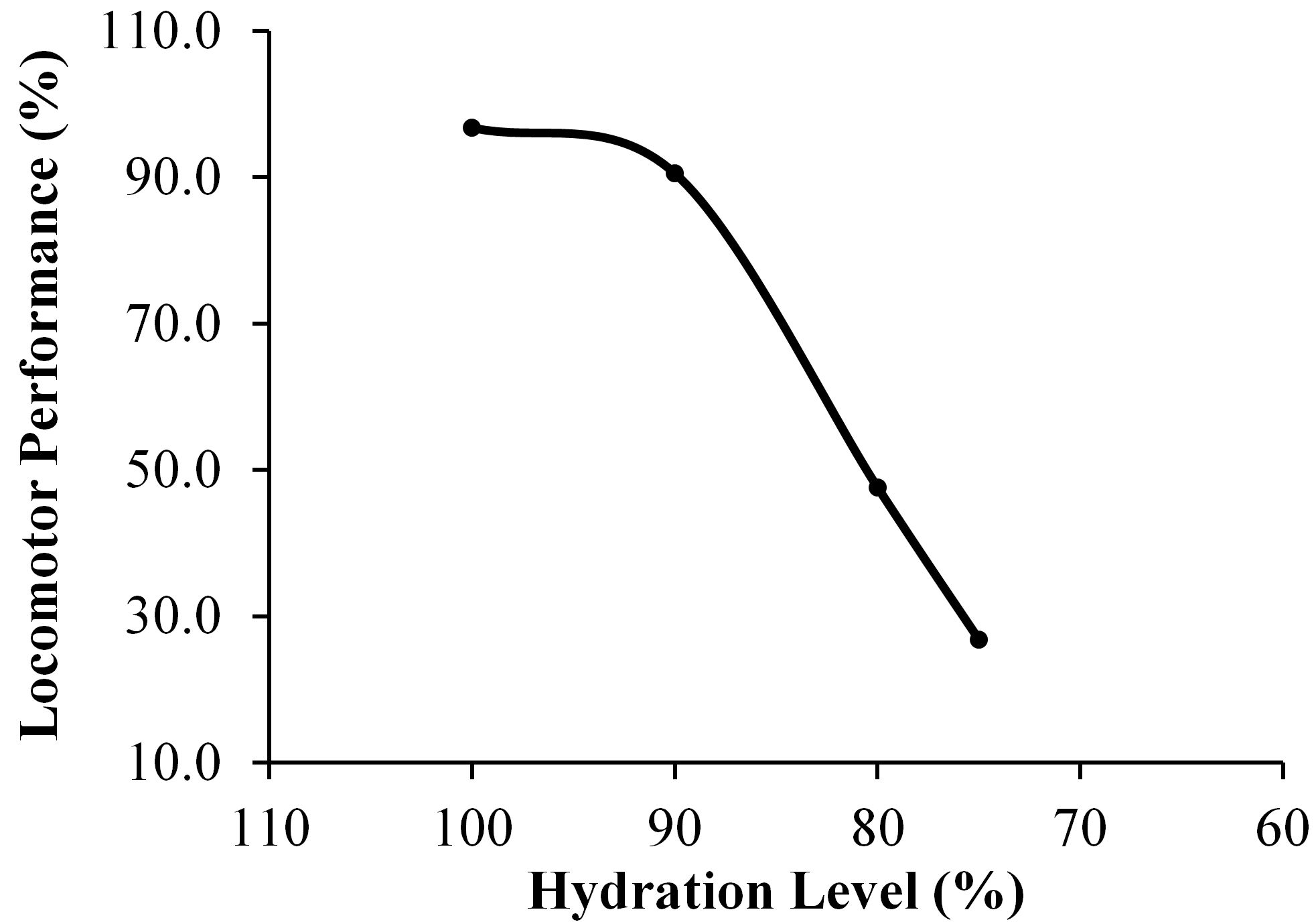

Supplement: S19 Fig — Mean locomotor performance transformed as a percentage of maximum performance in different hydration levels. (TIF) [file pone.0140761.s019.tif]

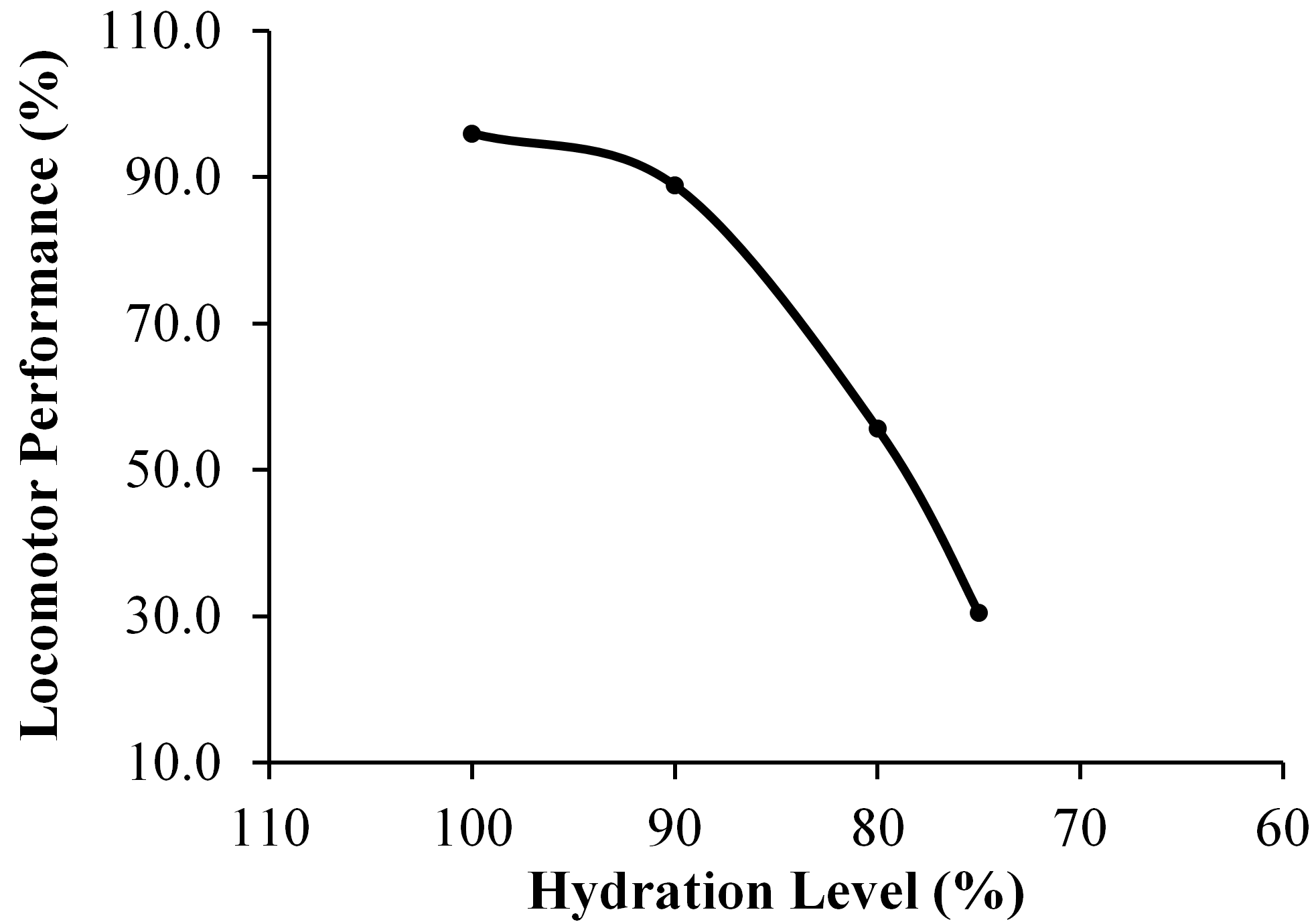

Supplement: S20 Fig — Mean locomotor performance transformed as a percentage of maximum performance in different hydration levels. (TIF) [file pone.0140761.s020.tif]

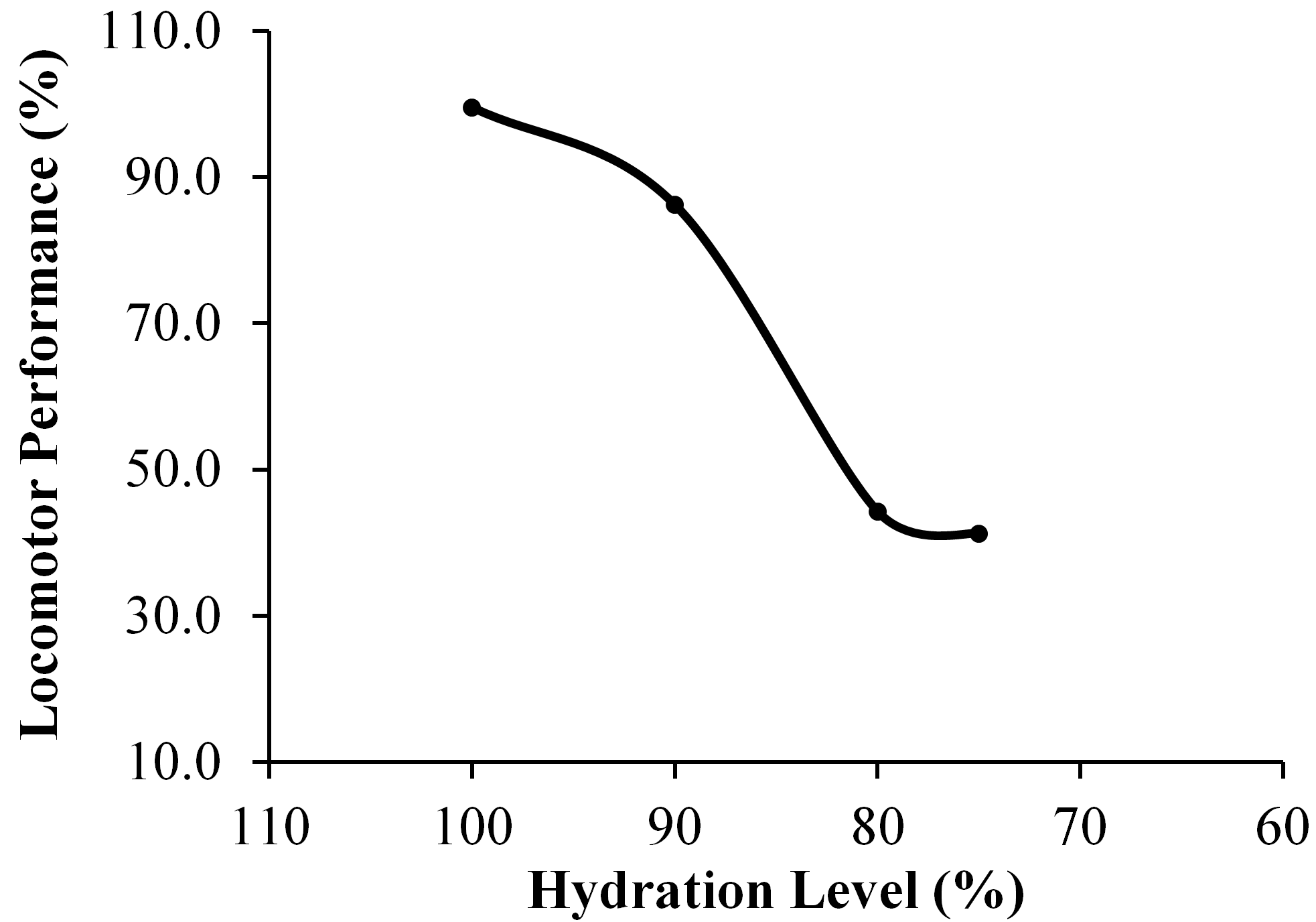

Supplement: S21 Fig — Mean locomotor performance transformed as a percentage of maximum performance in different hydration levels. (TIF) [file pone.0140761.s021.tif]

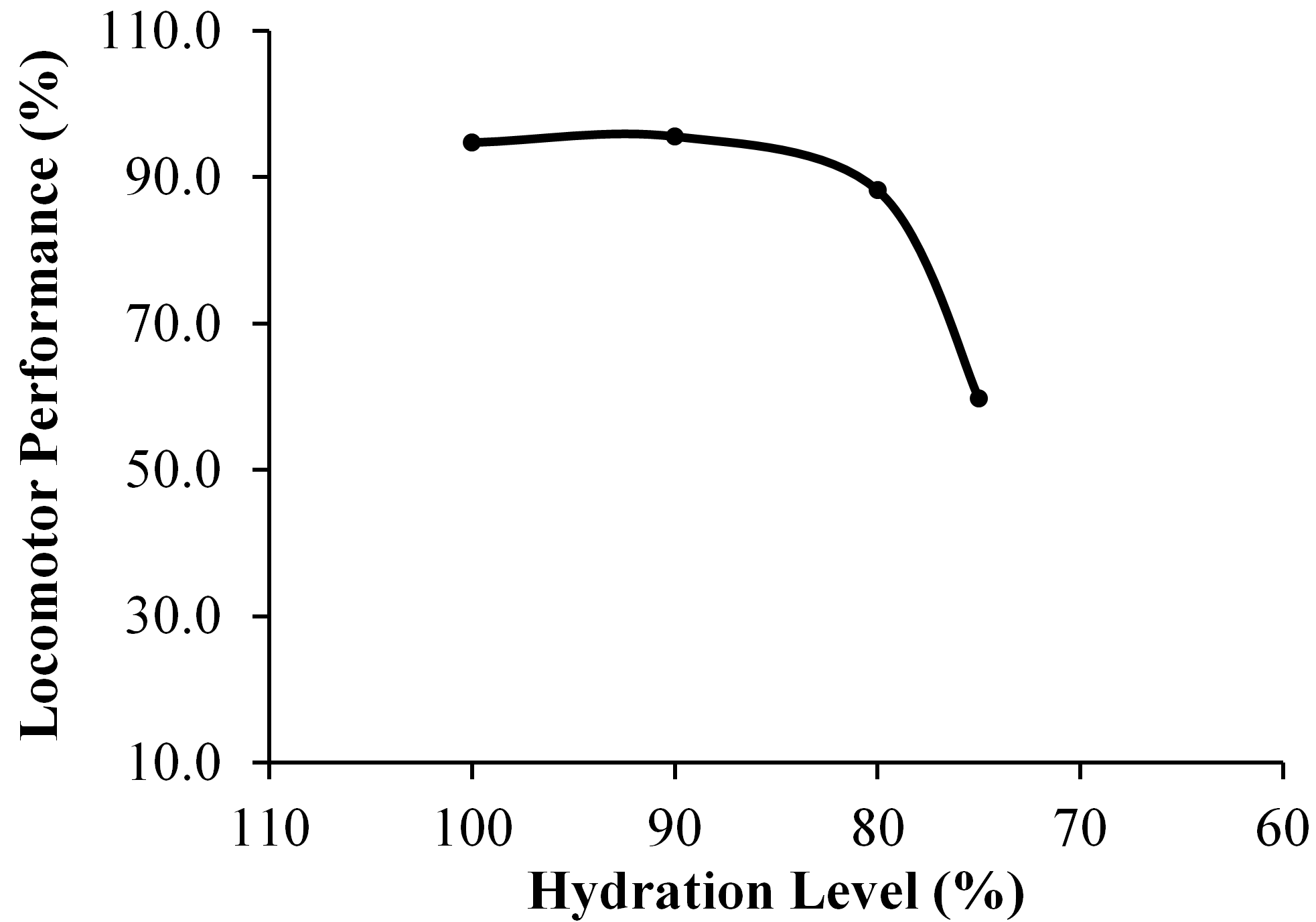

Supplement: S22 Fig — Mean locomotor performance transformed as a percentage of maximum performance in different hydration levels. (TIF) [file pone.0140761.s022.tif]

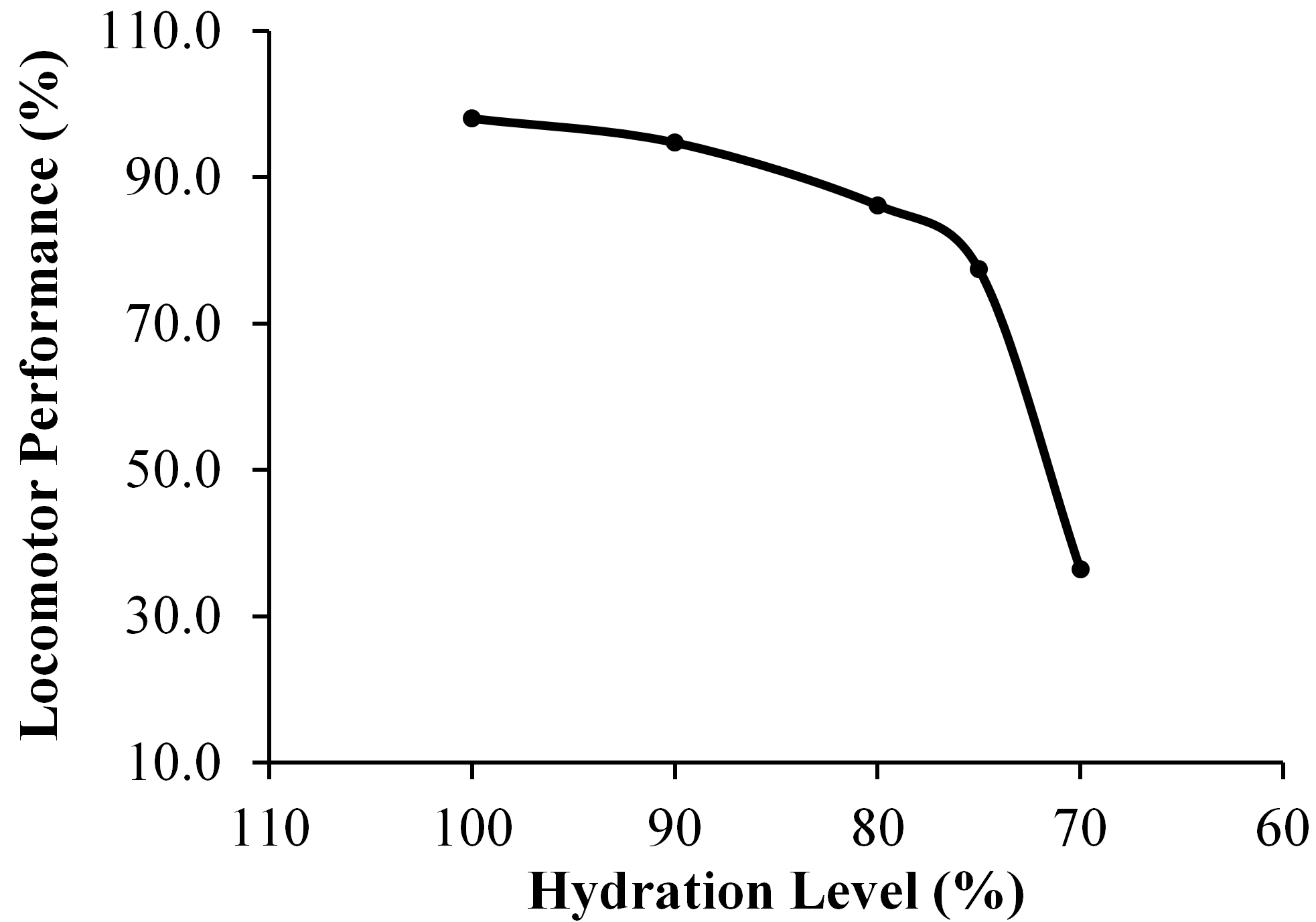

Supplement: S23 Fig — Mean locomotor performance transformed as a percentage of maximum performance in different hydration levels. (TIF) [file pone.0140761.s023.tif]

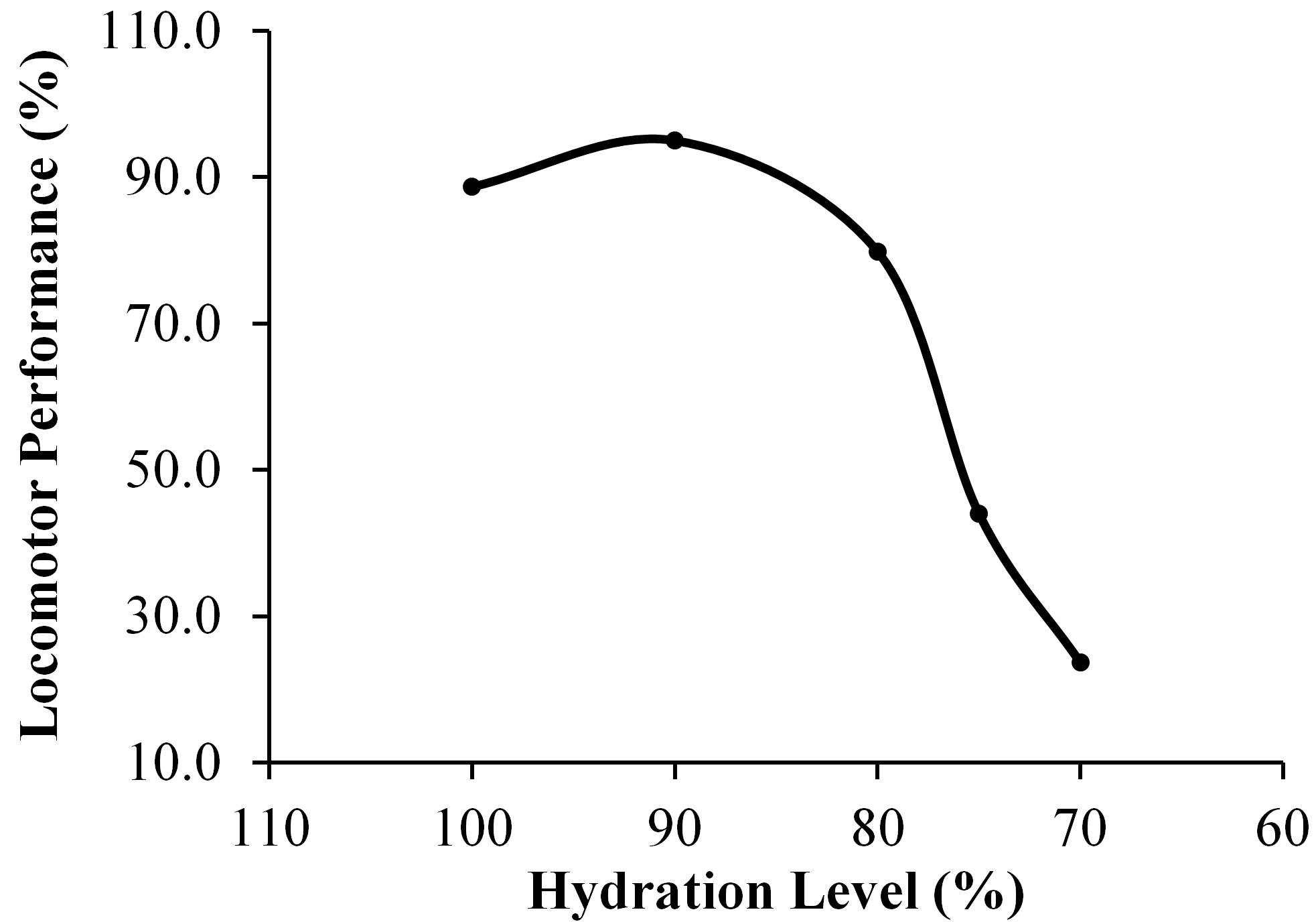

Supplement: S24 Fig — Mean locomotor performance transformed as a percentage of maximum performance in different hydration levels. (TIF) [file pone.0140761.s024.tif]

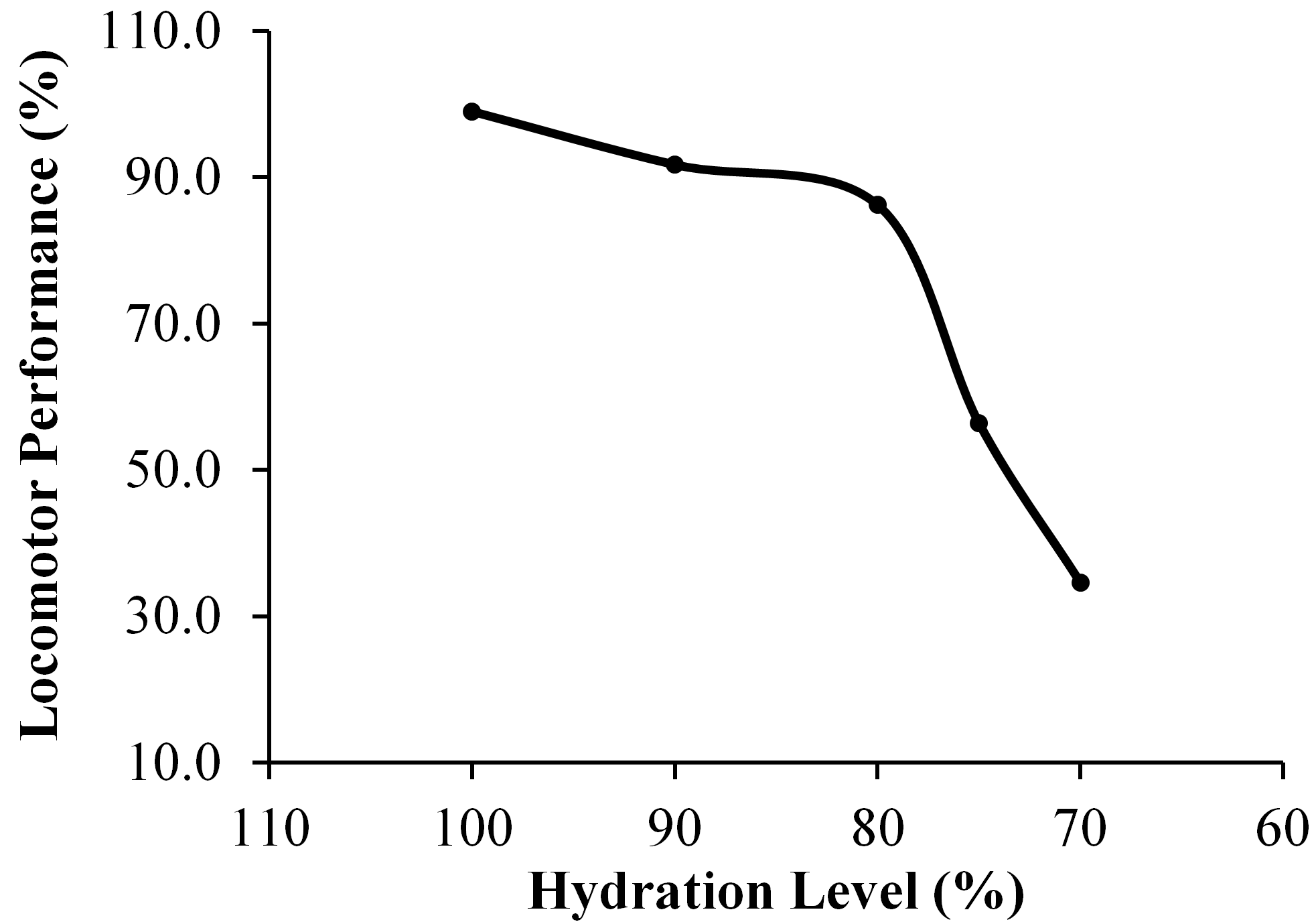

Supplement: S25 Fig — Mean locomotor performance transformed as a percentage of maximum performance in different hydration levels. (TIF) [file pone.0140761.s025.tif]

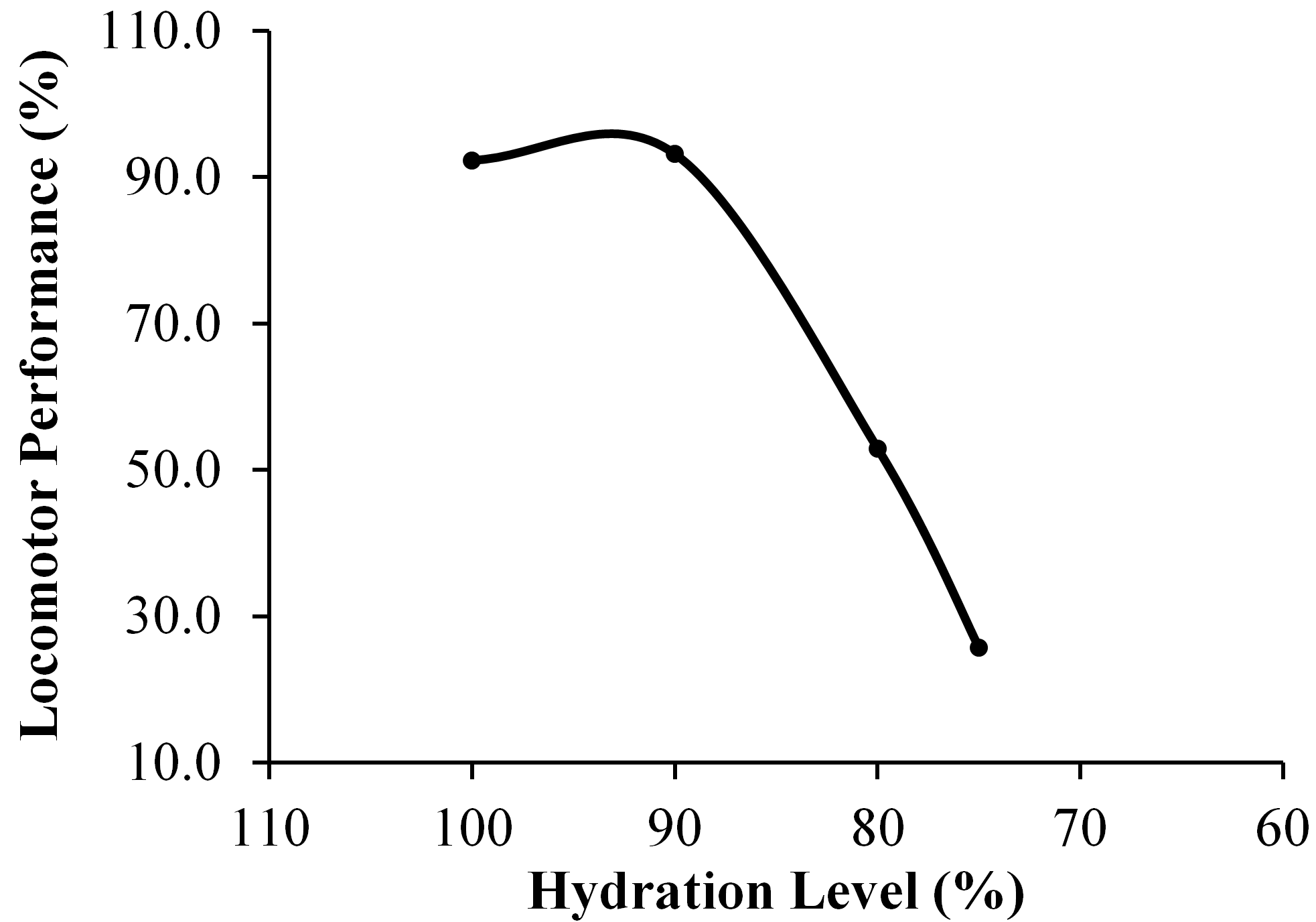

Supplement: S26 Fig — Mean locomotor performance transformed as a percentage of maximum performance in different hydration levels. (TIF) [file pone.0140761.s026.tif]

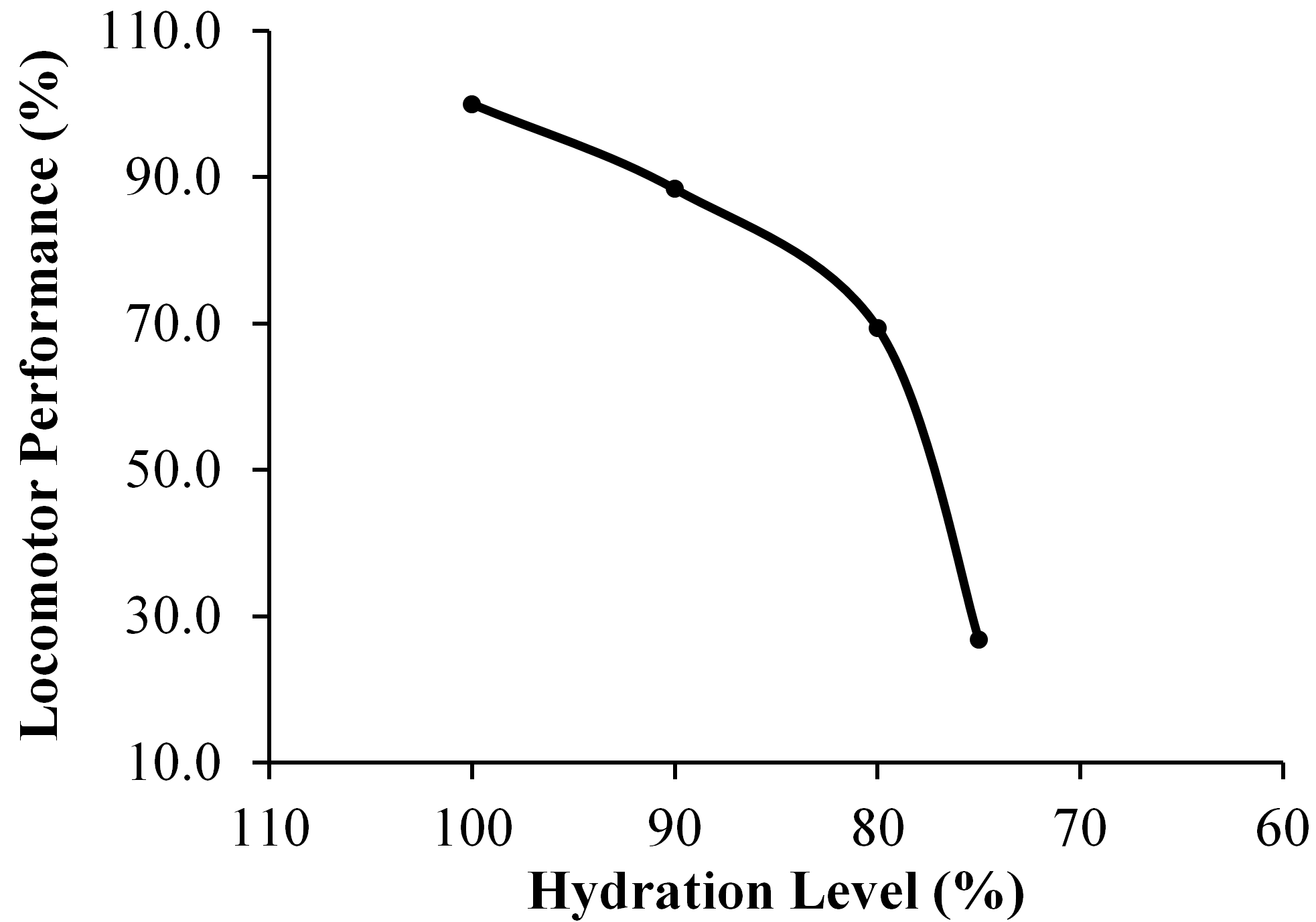

Supplement: S27 Fig — Mean locomotor performance transformed as a percentage of maximum performance in different hydration levels. (TIF) [file pone.0140761.s027.tif]

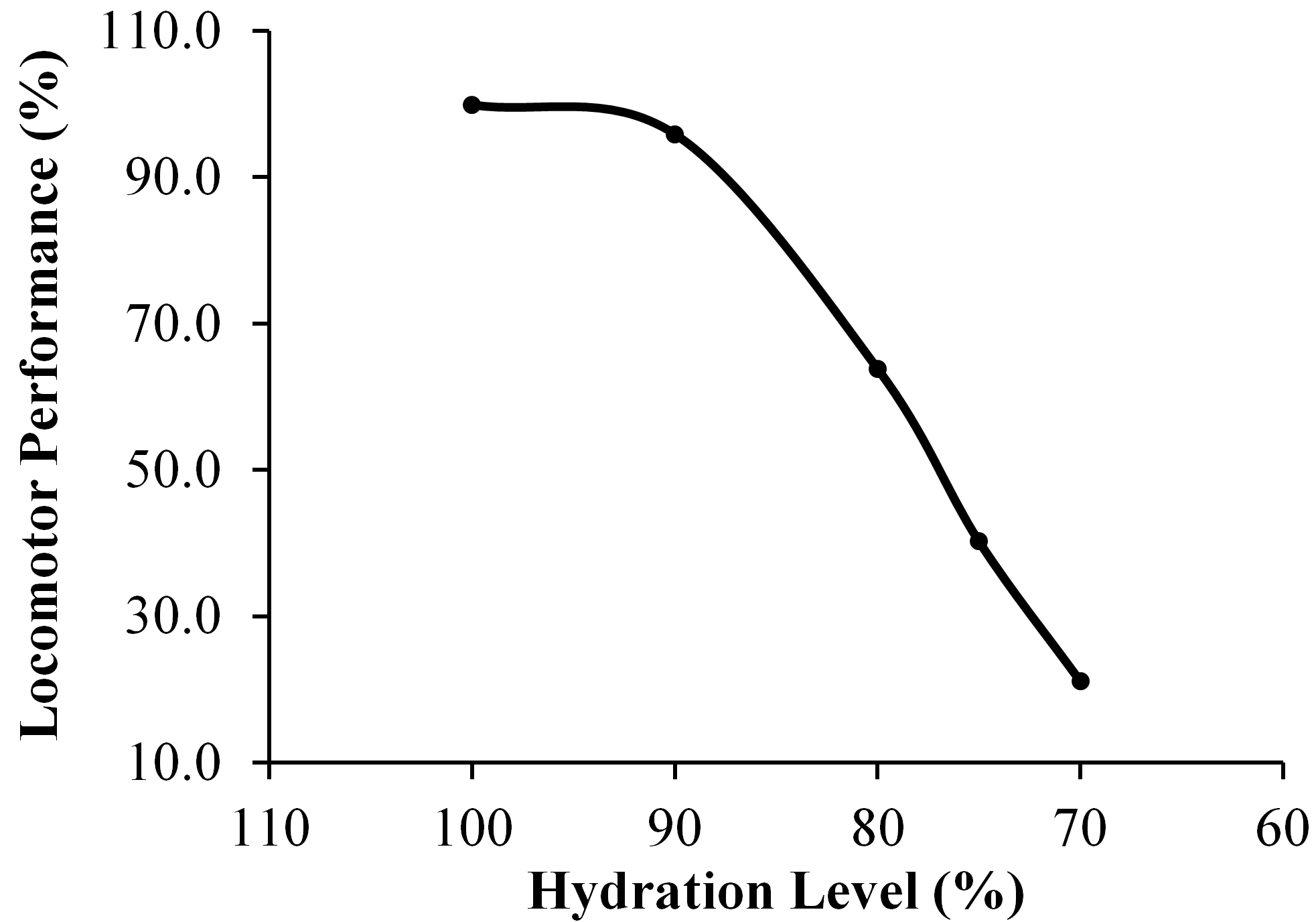

Supplement: S28 Fig — Mean locomotor performance transformed as a percentage of maximum performance in different hydration levels. (TIF) [file pone.0140761.s028.tif]

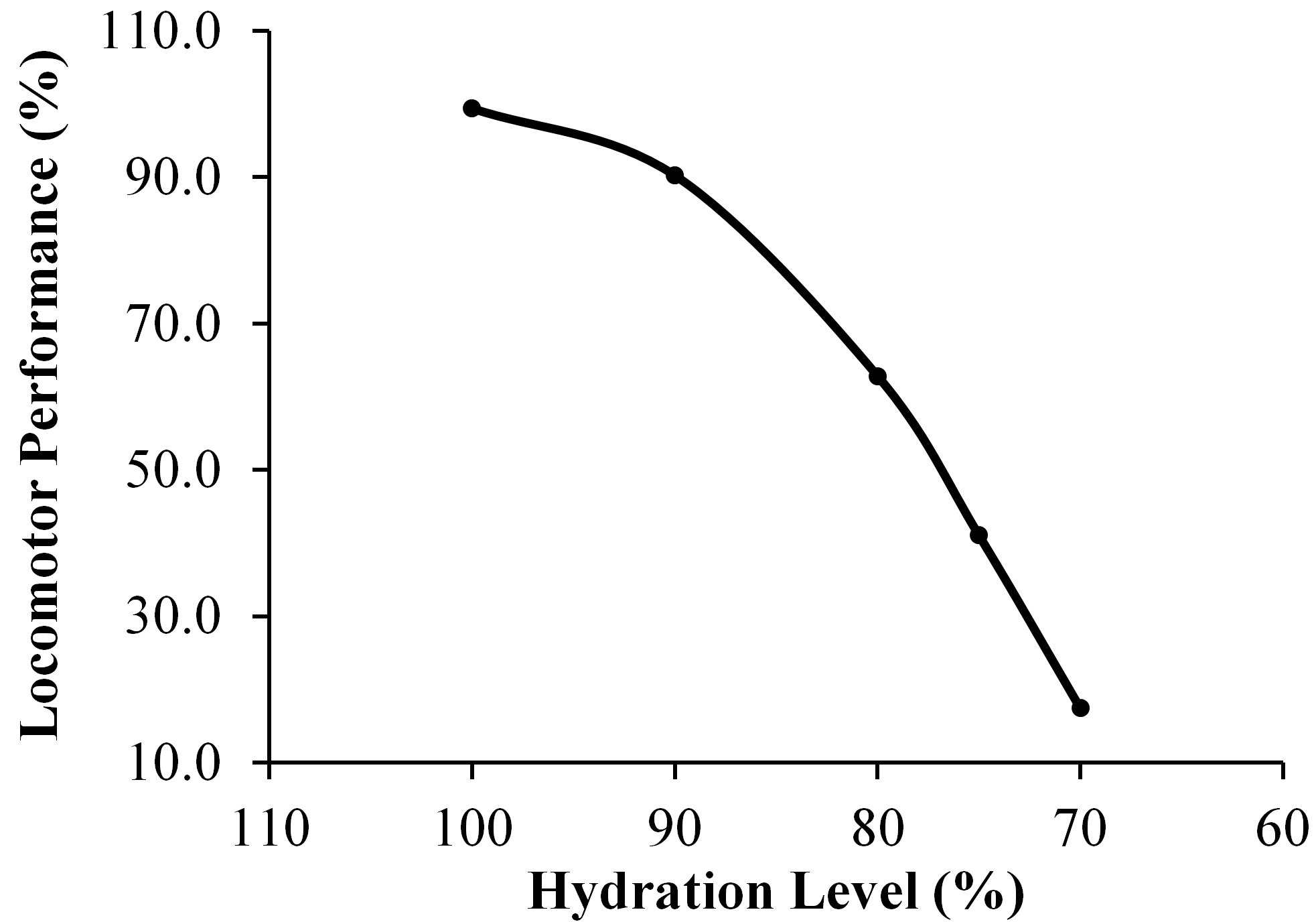

Supplement: S29 Fig — Mean locomotor performance transformed as a percentage of maximum performance in different hydration levels. (TIF) [file pone.0140761.s029.tif]

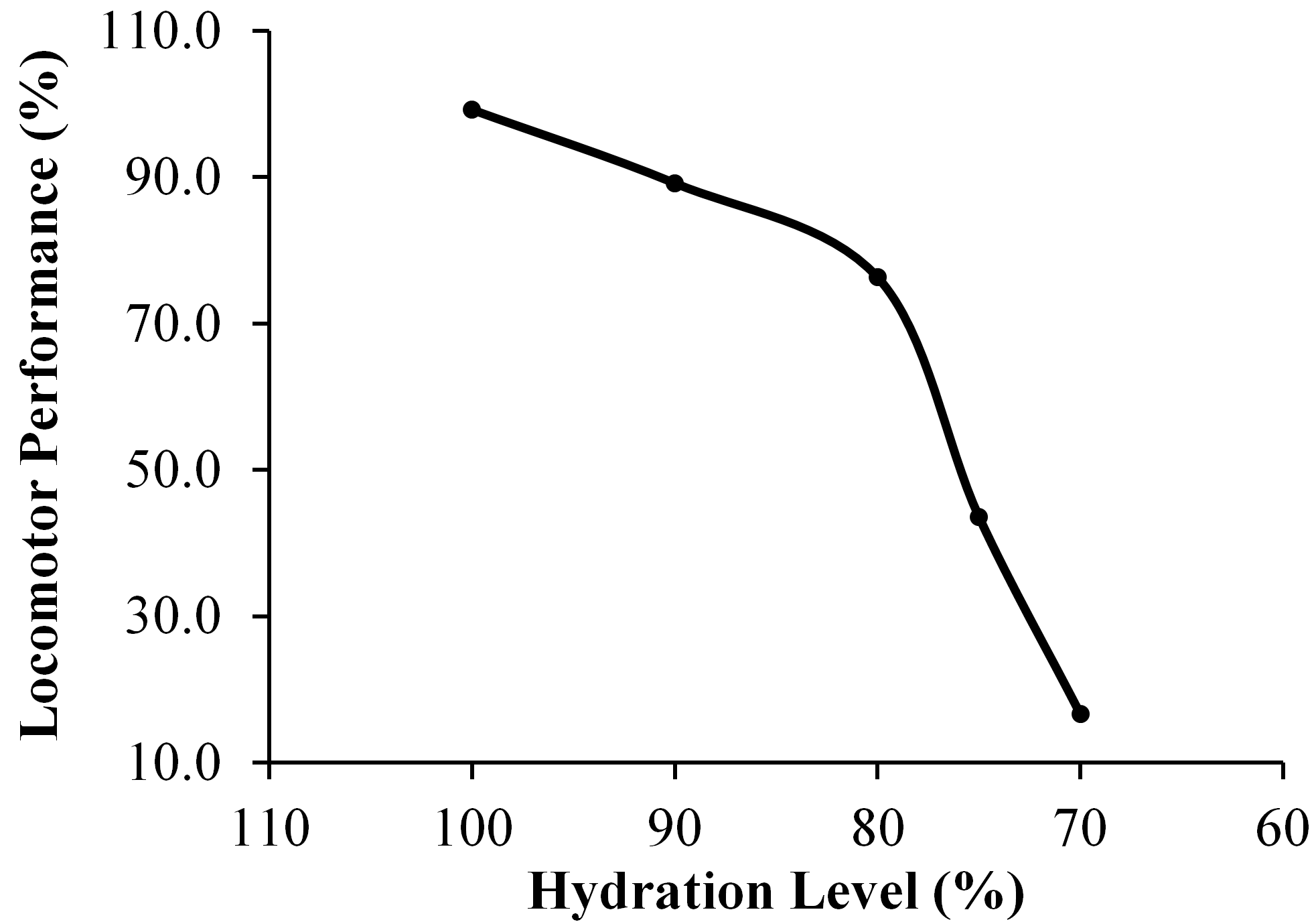

Supplement: S30 Fig — Mean locomotor performance transformed as a percentage of maximum performance in different hydration levels. (TIF) [file pone.0140761.s030.tif]
