# Supplementary material for: Relation between Water Balance and Climatic Variables Associated with the Geographical Distribution of Anurans
Source: PLoS One. 2015 Oct 15;10(10):e0140761. doi: 10.1371/journal.pone.0140761 (PMC4607303; doi:10.1371/journal.pone.0140761)

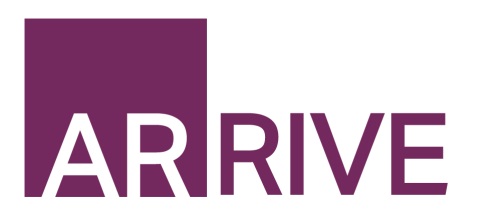


The ARRIVE Guidelines Checklist

Animal Research: Reporting In Vivo Experiments

Carol Kilkenny^1^, William J Browne^2^, Innes C Cuthill^3^, Michael Emerson^4^ and Douglas G Altman^5^

*^1^The National Centre for the Replacement, Refinement and Reduction of Animals in Research, London, UK, ^2^School of Veterinary Science, University of Bristol, Bristol, UK, ^3^School of Biological Sciences, University of Bristol, Bristol, UK, ^4^National Heart and Lung Institute, Imperial College London, UK, ^5^Centre for Statistics in Medicine, University of Oxford, Oxford, UK.*

|  | | ITEM | RECOMMENDATION | Section/ Paragraph |
| --- | --- | --- | --- | --- |
| 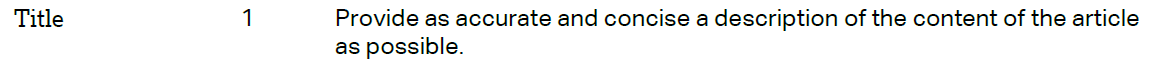 | | | Title |  |
| 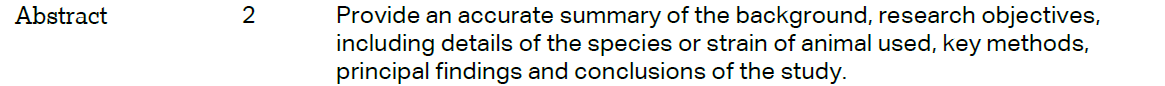 | | | Abstract |  |
| INTRODUCTION | | |  |  |
| 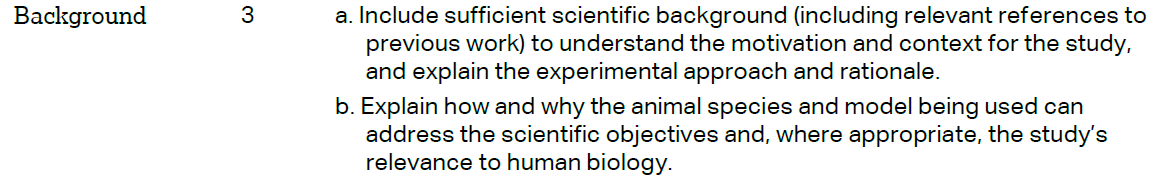 | | | Introduction,  Paragraph 1-3  Paragraph 3-4 |  |
| 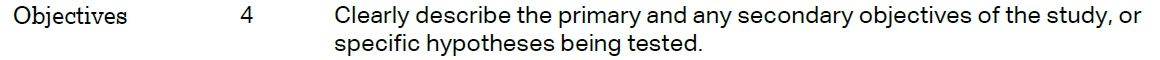 | | | Paragraph 5 |  |
| METHODS | | |  |  |
| 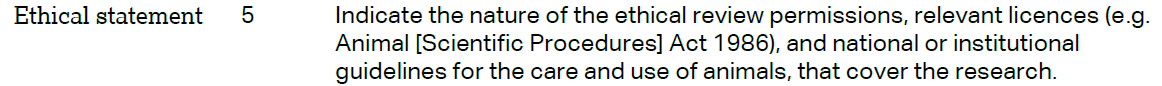 | | | Methods, subsection 1 “Collection Localities and Animal Maintenance”  Paragraph 1 |  |
| 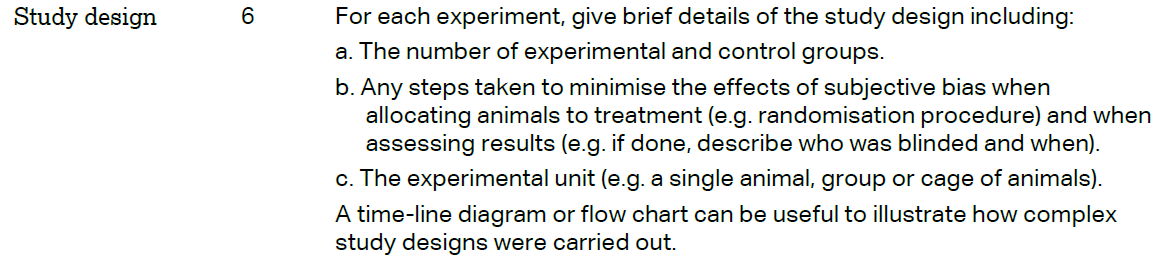 | | | Methods, subsection 2 “Sensitivity of locomotor performance to dehydration”  Paragraph 1 |  |
| 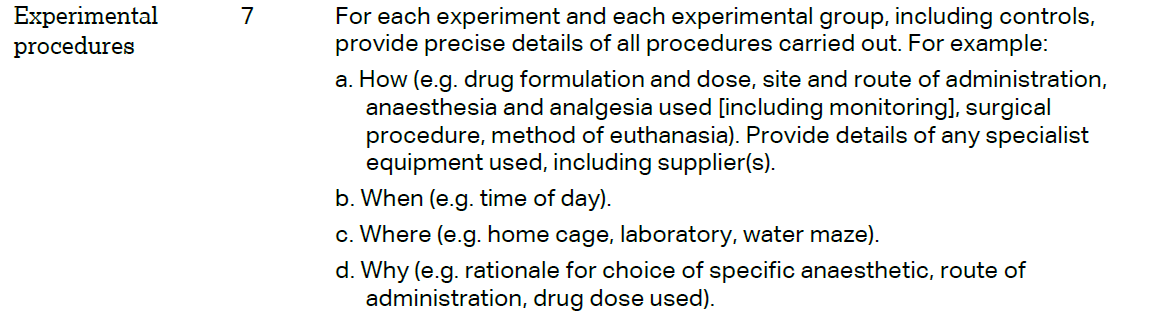 | | | Methods, subsection 2 “Sensitivity of locomotor performance to dehydration”  Paragraph 1 |  |
| 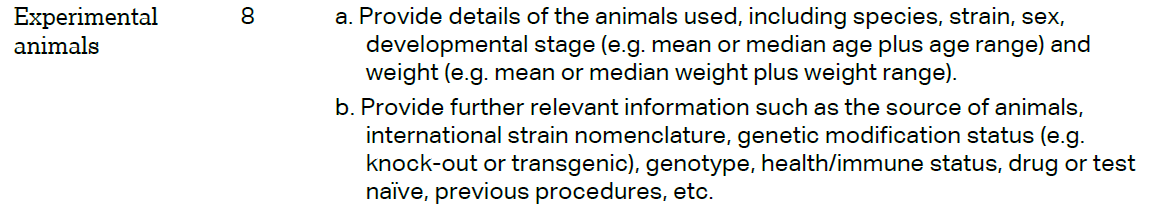 | | | Results, Table 1  Methods, subsection 1 “Collection Localities and Animal Maintenance”  Paragraph 1 |  |

The ARRIVE guidelines. Originally published in *PLoS Biology*, June 2010^1^

| 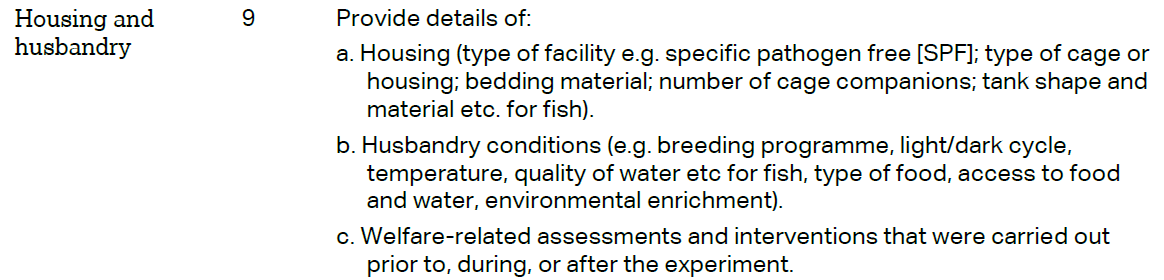 | Methods, subsection 1 “Collection Localities and Animal Maintenance”  Paragraph 1 | |
| --- | --- | --- |
| 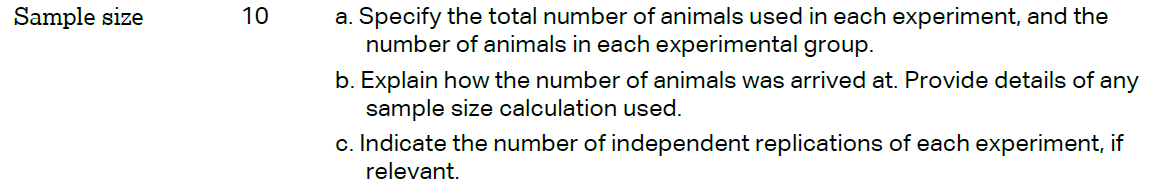 | Results, Table 1  Methods, subsection 2 “Sensitivity of locomotor performance to dehydration”  Paragraph 1 | |
| 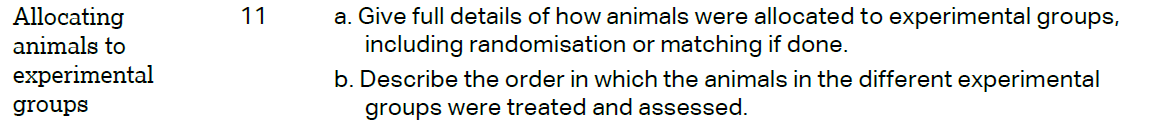 | Methods, subsection 2 “Sensitivity of locomotor performance to dehydration”  Paragraph 1 | |
| 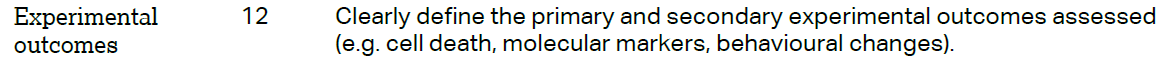 | Methods, subsection 2 “Sensitivity of locomotor performance to dehydration”  Paragraph 1 | |
| 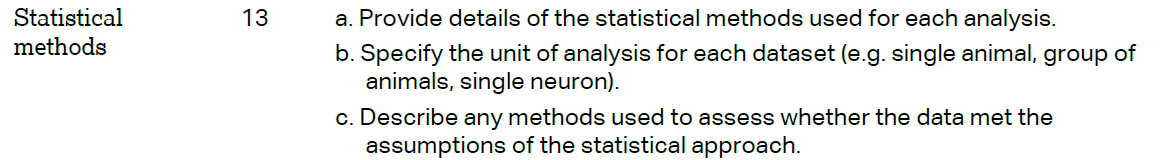 | Methods, subsection 7 “Statistical analyses”  Paragraph 1-3 | |
| RESULTS |  | |
| 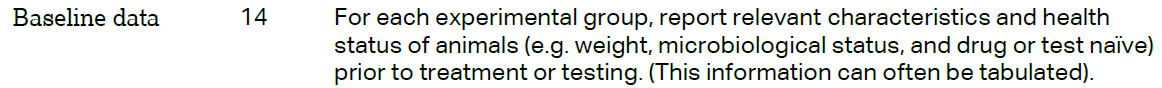 | Results, Table 1 | |
| 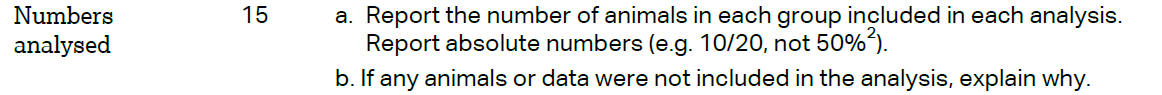 | Results, Table 1 | |
| 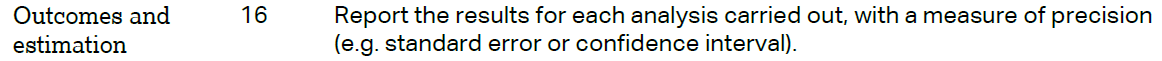 | Results, Table 1  subsection 2  “Allometry and relations between climate and physiological variables”  Paragraph 1, Table 5 and Figure 2 and 3. | |
| 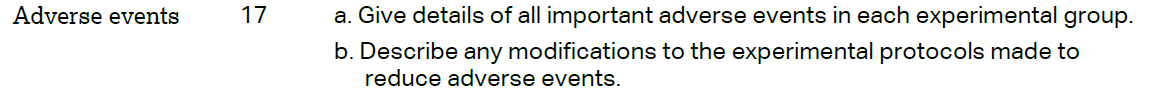 | Not applicable. | |
| DISCUSSION |  | |
| 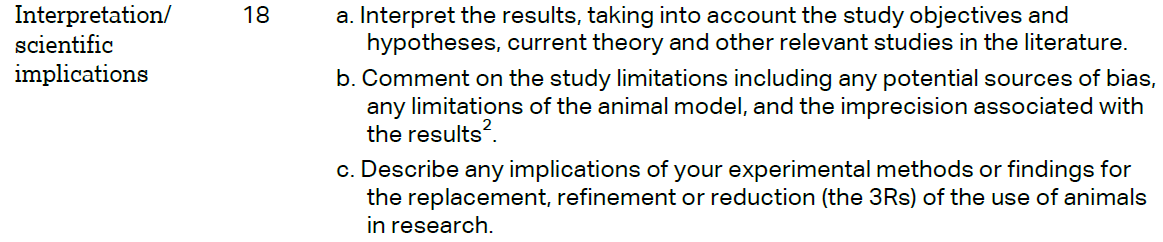 | All paragraphs of Discussion section | |
| 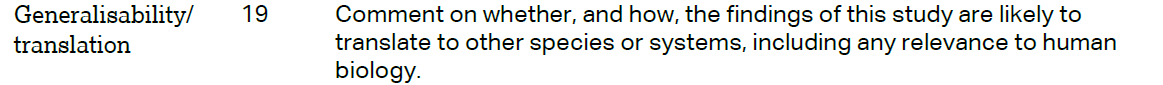 | Discussion,  Paragraphs 3-5 | |
| 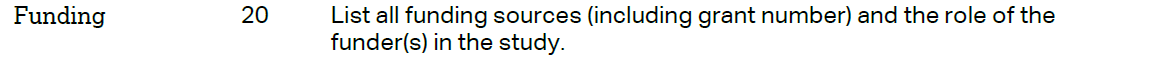 | | Instructed to not include in manuscript file.  Full information included during submission on “Additional Information” section at the topic “Financial Disclosure” |


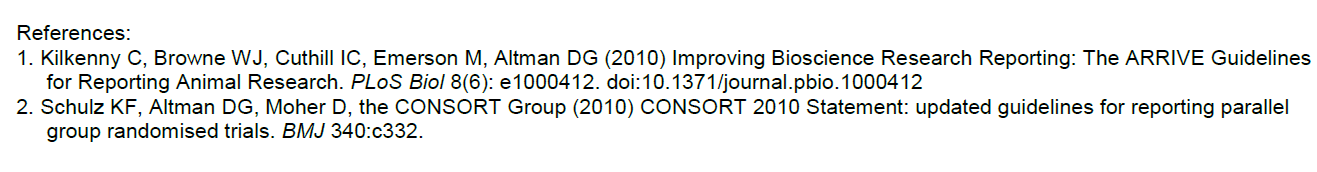

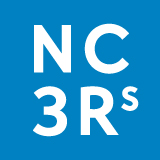

Supplement: S1 Text — Animal Research: Reporting In Vivo Experiments Guidelines Checklist. (DOCX) [file pone.0140761.s031.docx]
